# Host-Associated Binding Patterns in Predicted Structural Models of Circovirus Capsid–Importin Complexes

**DOI:** 10.34133/csbj.0253

**Published:** 2026-11-06

**Authors:** Yue Wang, Junping Wen, Jipei Zhang, Shujun Yang, Cuicui Meng, Bingyu Yu, Yitong Ye, Yinsheng Lin, Wenchang Xue, Jinling Huang, Jidang Chen, Tong Wang

**Affiliations:** ^1^ School of Animal Science and Technology, Foshan University, Foshan 528225, Guangdong, China.; ^2^ Department of Emergency, The Eighth Affiliated Hospital of Sun Yat-sen University, Shenzhen 518033, Guangdong, China.; ^3^ Agricultural Teaching and Research Base, Foshan University, Foshan 528225, Guangdong, China.

## Abstract

The nuclear entry of the capsid protein is a critical step in the life cycle of circoviruses, but whether capsid proteins from different circoviruses differ in their importin interactions remained unclear. Here, we modeled 298 unique circovirus capsid protein–importin pairs using AlphaFold 3. After conformational clustering and ZDOCK concordance filtering, 603 representative interfaces were retained for analysis. We extracted Δ*iG* values, interface features, and nuclear localization signal (NLS)-specific interface properties by PDBePISA and analyzed them with statistical tests. Capsid protein (Cap)–importin-9 (IPO9) complexes showed the lowest mean Δ*iG* among all tested circoviruses, identifying IPO9 as a candidate broadly interacting importin among the viruses examined. Across all host classes, Cap–importin β complexes showed a lower mean Δ*iG* than Cap–importin α or Cap–transportin complexes. Predicted Δ*iG* patterns differed across host classes, with distinct profiles for avian, mammalian, and fish circoviruses. Predicted Δ*iG* patterns and NLS interface features, including basic residue ratio, interface occupancy, and net charge, differed across avian, mammalian, and fish circoviruses. Together, these results provide a set of testable predictions regarding circovirus Cap–importin interfaces and highlight the predicted NLS interface as a region for further investigation.

## Introduction

Circoviruses represent some of the smallest known DNA viruses, harboring circular single-stranded genomes of approximately 1.7 to 2.0 kb. Despite this genetic simplicity, they can infect a wide range of vertebrate hosts, including birds, mammals, and fish [1,2]. Although these viruses are very simple in structure, why they can infect so many species remains poorly understood.

Circoviruses cause serious disease in domestic animals and lead to considerable economic losses. Porcine circovirus type 2 (PCV2), for example, is a fatal swine pathogen that causes postweaning multisystemic wasting syndrome [3]. Beak and feather disease virus (BFDV) can cause severe disease in parrots, threatening the survival of endangered psittacine species. Human circovirus 1 (HCV1) was recently identified in humans [4,5].

Because circoviruses replicate their DNA in the host cell nucleus, the capsid protein (Cap) monomers are presumed to be transported into the nucleus to interact with the replicated viral genome. Passive diffusion through the nuclear pore complex is generally considered efficient for small molecules, while larger proteins can also diffuse slowly and size alone does not strictly determine permeability [6,7]. Circovirus capsid proteins, although typically only 25 to 35 kDa as monomers, require active nuclear transport [8].

Protein nuclear transport is mediated by importins, which recognize nuclear localization signals (NLSs) on cargo proteins [9]. In the classical import pathway, importin α binds to KPNB1 via its N-terminal IBB (importin β-binding) domain, and the heterodimer recognizes cargo proteins carrying classical NLS motifs, therefore enabling their translocation into the nucleus [10–12]. The importin β family includes receptors that can bind cargo directly, without requiring importin α, and mediate nuclear import through interaction with the nuclear pore complex and RanGTP-dependent cargo release [13]. The transportin family also belongs to the importin β superfamily and recognizes linear motifs. TNPO1 and TNPO2 recognize the PY-NLS motif, whereas TNPO3 recognizes RS/RSY-rich motifs [14,15]. Therefore, they are treated as a separate operational category in this study.

Anelloviruses, a family of single-stranded DNA viruses evolutionarily related to circoviruses, share a conserved capsid fold with circoviruses. Their capsid protein (ORF1) contains an N-terminal arginine-rich region that is structurally and sequentially similar to the NLS of circovirus capsids [16–18]. Interestingly, anelloviruses have also acquired an additional classical NLS at the C-terminus, which has been proposed to compensate for the progressive burial of the N-terminal NLS during capsid assembly [19]. Whether similar structural constraints affect NLS presentation and its accessibility for importin binding in circoviruses remains unclear.

Previous experimental work has shown that the PCV2 Cap interacts with multiple importins, including importin α, IPO4, IPO5, IPO7, and IPO11, and its NLS regulates nuclear import and export through phosphorylation [20]. For PCV2 and BFDV, basic regions within the capsid proteins have been identified as putative NLS motifs and shown to be associated with nuclear localization [21–23]. However, previous studies on circovirus nuclear import are mostly limited to individual species, and whether Cap–importin binding patterns differ across host classes remains unexplored.

To address these questions, we combined structural modeling with interface feature extraction and statistical comparison. We modeled 298 unique Cap–importin pairs, covering 11 circovirus species and 18 host importins from 3 operational groups. AlphaFold 3, as implemented on the AlphaFold Server (hereinafter referred to as AF3), was used as the primary modeling method, and ZDOCK was employed for concordance filtering of the predicted binding modes.

We then used statistical analyses to compare predicted Cap–importin binding patterns across host classes and to examine whether NLS interface characteristics differ across host classes. In addition, we performed structural comparisons of representative circovirus and anellovirus Cap–importin binding modes to further explore the structural basis of these patterns. Together, these analyses provide a set of testable hypotheses regarding host-associated differences in predicted Cap–importin binding.

## Materials and Methods

### Data collection

Capsid protein (Cap) sequences from 11 circovirus species spanning 3 host classes were retrieved from the National Center for Biotechnology Information (NCBI) GenBank database. The dataset comprised 4 avian viruses, namely, goose circovirus (GoCV; NP_150370.1), pigeon circovirus (PiCV; NP_059530.1), BFDV (AKP49411.1), and duck circovirus (DuCV; NP_877979.1). Four mammalian viruses were included: PCV2 (NP_937957.1), porcine circovirus type 3 (PCV3; WYC12412.1), bat circovirus (BCV; YP_010084305.1), and HCV1 (YP_013607441.1). Three fish viruses were also included: eel circovirus (ECV; APZ87909.1), catfish circovirus (CCV; YP_009091697.1), and bream circovirus 1 (BCV1; XCD31261.1). Species were selected to balance representation across host classes while ensuring that complete importin sequences could be retrieved from each corresponding natural host. Additionally, the capsid protein sequence of torque teno sus virus (TTSV; QKE51843.1), a representative anellovirus, was included for structural comparison with circovirus capsids (“Structural features of predicted NLS residues in Cap–importin complexes” section).

For each virus, importin sequences from its natural host species were collected from NCBI GenBank, covering 3 operational groups. These were the importin α group (7 members: KPNA1 to KPNA7), the importin β group (8 members: KPNB1, IPO4, IPO5, IPO7, IPO8, IPO9, IPO11, and IPO13), and the transportin group (3 members: TNPO1 to TNPO3). Where available, additional isoforms or subtypes were also included, yielding a total of 298 unique Cap–importin combinations. The full list of importin accession numbers is provided in Data File S2.

### Structural modeling and feature extraction

For each Cap–importin pair, the sequences of the capsid protein and the corresponding host importin were submitted to the AlphaFold Server [24]. Five ranked structural models were retrieved per pair. To account for potential conformational variability, the binding interfaces of the 5 AF3 models were compared pairwise using DockQ v2, with the Critical Assessment of PRedicted Interactions (CAPRI) “acceptable” threshold used to determine whether 2 models shared a similar binding mode [25,26]. Models that did not meet this threshold were grouped as distinct conformational clusters. For each identified cluster, the top-ranked AF3 model (by confidence score) was selected as the representative structure. The acceptable threshold was used for clustering to avoid oversplitting of minor conformational variants. AF3 confidence metrics, including predicted local distance difference test, predicted template modeling score, interface predicted template modeling score, and interface predicted aligned error (iPAE), are summarized in Table S1. iPAE was calculated as the mean predicted aligned error between predicted NLS residues (“NLS sequence prediction and feature engineering” section) and importin interface residues, with interface residues defined by a 5-Å heavy-atom distance cutoff [27]. Values for each of the 603 AF3 models are provided in Data File S1. AF3 predictions were performed using the AlphaFold Server (dialect: alphafoldserver, version 1), with runs performed between December 2025 and August 2026. Individual run dates are recorded in the corresponding job_request.json files deposited in the data repository.

To filter AF3-predicted binding modes by concordance, each representative AF3 model was separated into its monomeric components (Cap and importin) and submitted as separate receptor and ligand structures to the ZDOCK server [28]. Rigid-body docking was performed using ZDOCK 3.0.2 with default settings on the ZDOCK server, and the top 10 docking poses were retained [29]. Each AF3 representative model was then compared against the corresponding top 10 ZDOCK poses by interface alignment. The CAPRI “acceptable” quality threshold was applied to identify the ZDOCK poses that most closely resembled the AF3-predicted binding mode. The best-matching ZDOCK model (or the one that met the acceptable criterion) was retained as a second representation of the corresponding AF3 model interface. If no ZDOCK pose reached the acceptable threshold for a given AF3 representative, the corresponding AF3 model was considered not reproducible by rigid-body docking and was excluded from subsequent analyses. The scripts used for AF3 model clustering, DockQ scoring, ZDOCK concordance filtering, and CAPRI-based filtering are provided in File S1. The number of conformational clusters retained and excluded by ZDOCK concordance filtering is summarized by virus in Table S2 and by importin group in Table S3.

Through this workflow, the initial 298 Cap–importin pairs were expanded to 707 conformational clusters. After ZDOCK concordance filtering, 603 clusters passed and 104 failed, yielding a total of 1,206 models—603 from AF3 and 603 from ZDOCK (Fig. S1). All models were subsequently analyzed using PDBePISA to extract interface properties [30]. Δ*iG* (the solvation free energy gain upon interface formation, kcal/mol), interface area (solvent-accessible surface area buried at the interface, Å^2^), hydrogen bond count, and salt bridge count were extracted. Derived features included Δ*iG* per interface area, hydrogen bond density (H-bonds divided by interface area), salt bridge density (salt bridges divided by interface area), and the hydrogen bond-to-salt bridge ratio. To check the dynamic stability of the predicted binding modes, 50-ns molecular dynamics simulations were performed on 5 representative Cap–importin complexes [31,32]. Full simulation parameters are provided in Supplementary Methods Section 1.1, and results are presented in Figs. S2 to S6. The complete dataset of all 1,206 models is provided in Data File S1.

As a control for the autoinhibitory IBB domain of importin α, we additionally modeled PCV2 Cap in complex with ΔIBB importin α constructs (IBB domain removed) across all importin α isoforms. These complexes were processed through the same AF3–ZDOCK–PDBePISA pipeline, and Δ*iG* values were compared between full-length and ΔIBB constructs at the pair level. The complete dataset is provided in Data File S3.

### Experimental validation of Cap–IPO9 interactions

To experimentally validate the predicted Cap–importin-9 (IPO9) interactions, we performed co-immunoprecipitation (Co-IP) assays for 3 representative virus–host pairs, one from each host class: GoCV (avian), HCV1 (mammalian), and CCV (fish). The corresponding host IPO9 sequences were from goose (XP_066839304.1), human (EAW91377.1), and catfish (XP_046730955.1), respectively.

CHO-K1 cells were cultured in Dulbecco’s modified Eagle medium/nutrient mixture F-12 (DMEM/F12) medium supplemented with 10% fetal bovine serum at 37 °C and 5% CO_2_. Cells were co-transfected with pcaggs-Cap-His and pcaggs-Flag-IPO9 using Lipofectamine 3000 (Invitrogen). At 72 h posttransfection, cells were harvested and lysed in lysis buffer containing 150 mM NaCl, 1.0% Triton X-100, 0.5% sodium deoxycholate, and 50 mM Tris.

For Co-IP, cell lysates were incubated with BeyoMag Anti-Flag Magnetic Beads (Beyotime, P2181) at 4 °C overnight. After washing, bound proteins were eluted and analyzed by Western blotting. Cap-His was detected with rabbit anti-6×His tag antibody (Abcam, ab9108), and Flag-IPO9 was detected with rabbit anti-DDDDK tag antibody [EPR20018-251] (Abcam, ab205606), followed by goat anti-rabbit IgG H&L (horseradish peroxidase) secondary antibody (Abcam, ab6712). As a negative control, cells transfected with pcaggs-Cap-His alone were subjected to the same anti-Flag immunoprecipitation. Each Co-IP experiment was performed in 3 independent biological replicates.

### Evolutionary conservation analysis

IPO9 sequences from the 10 host species listed above were aligned using MAFFT with default parameters [33,34]. Conservation scores were calculated as the frequency of the most common amino acid at each position, ranging from 0 (completely variable) to 1 (completely conserved). A sliding window of 10 amino acids was applied to generate the conservation profile. Conserved regions (CR) were defined as stretches of ≥10 consecutive residues with conservation scores ≥0.9. Multiple sequence alignment and evolutionary conservation analysis were performed using the script in File S1. This analysis was performed as a descriptive, supplementary analysis to contextualize the predicted Cap–IPO9 interfaces and was not used to draw functional or evolutionary conclusions.

### Statistical analysis

All statistical analyses were performed in Python 3.10.12 using SciPy 1.15.3 and statsmodels 0.14.6.

The statistical unit was defined as the unique Cap sequence–importin sequence combination. AF3 and ZDOCK gave comparable Δ*iG* values for the same conformation in a paired comparison (mean paired difference = 0.22 kcal/mol, Wilcoxon signed-rank test, *P* = 0.126, *n* = 603; Fig. S7). For each combination, the AF3 and ZDOCK models of the same conformation were first averaged, and the resulting conformation-level values were then averaged across conformations, yielding a single value per unique Cap–importin combination. All subsequent tests used these combination-level values. Comparisons were performed at this level, either within host classes (avian, mammalian, and fish) or within importin groups (importin α, importin β, and transportin), as indicated.

The normality of the Δ*iG* distribution was assessed using the Shapiro–Wilk, D’Agostino–Pearson, Anderson–Darling, and Kolmogorov–Smirnov tests (Fig. S8). The results were mixed, with 3 of the 4 tests indicating significant deviation from normality. The distribution showed mild negative skewness (−0.24) and slightly low kurtosis (−0.16), suggesting an approximately symmetric but formally nonnormal distribution. Nonparametric tests were therefore used for all downstream analyses.

For hypothesis testing, the Mann–Whitney *U* test was used for 2-group comparisons. For each comparison involving 3 groups, 3 pairwise tests were performed, and the Bonferroni-adjusted significance threshold (0.05/3 = 0.0167) was used. Because multiple Cap–importin combinations share the same Cap sequence or the same importin, the *P* values reported for host-class and importin-group comparisons should be interpreted as exploratory rather than as definitive tests of significance. Virus-level and importin-level sensitivity analyses are provided in Figs. S11, S12, and S14.

### NLS sequence prediction and feature engineering

To identify NLS regions in circovirus capsid proteins, we used NLStradamus, a hidden Markov model-based predictor that is not restricted to classical NLS motifs, making it suitable for the diverse NLS architectures expected in circoviruses [35]. NLS boundaries were taken from the Viterbi decoding output of NLStradamus. NLS prediction was performed independently of structural modeling; predicted NLS regions were not used to generate or constrain the AF3 models.

After complex prediction, predicted NLS residues were mapped onto the modeled interfaces. We intersected the NLStradamus-predicted NLS regions with the binding interfaces extracted by PDBePISA from the final set of validated models (as described in the “Structural modeling and feature extraction” section). For each interface, we calculated a set of NLS-specific features: the number of NLS residues at the interface, the net charge of these residues, the proportion of basic residues (lysine or arginine) among them, the number of salt bridges and hydrogen bonds involving NLS residues, the proportion of total salt bridges and hydrogen bonds contributed by NLS residues, the NLS interface occupancy ratio (i.e., NLS interface residues divided by total interface residues), and the NLS bond contribution ratio, defined as (NLS_HB + NLS_SB)/(HB + SB). A representative visualization of this mapping for Cap–IPO9 complexes is shown in Fig. S9. These features were subsequently used as inputs for machine learning (“Machine learning analysis” section).

In this study, NLS regions were treated solely as sequence-annotated residue ranges within the full-length Cap protein. No isolated NLS peptide was modeled, and no NLS-derived structure was used as a modeling input. All structural models were generated from full-length Cap and full-length importin sequences. “NLS interface residues” and all NLS-related features in the dataset refer to predicted NLS residues that are present at the modeled Cap–importin interface, not to all residues within the full predicted NLS region.

### Machine learning analysis

To examine how NLS interface features relate to host-class discrimination, we trained a random forest classifier (scikit-learn 1.7.2; 500 trees, minimum samples split = 5, balanced class weights) on the full set of 298 unique Cap–importin combinations. The input feature set included both the general interface features described in the “Structural modeling and feature extraction” section (Δ*iG*, interface area, hydrogen bond count, salt bridge count, and their derived densities) and the NLS-specific interface features described in the “NLS sequence prediction and feature engineering” section (NLS interface residues, NLS net charge, NLS basic residue ratio, NLS interface occupancy, NLS bond contribution, NLS hydrogen bonds, and NLS salt bridges). All features listed in the “NLS sequence prediction and feature engineering” section were used. Linear and polynomial regression did not capture the relationship between NLS features and host class, so a tree-based model was used to allow for nonlinear associations.

Shapley additive explanations (SHAP) dependence plots with locally weighted scatterplot smoothing were generated to visualize the direction and nonlinearity of the relationship between individual NLS features and host-class prediction. Feature importance and SHAP values were computed on the full dataset and are reported as in-sample exploratory analyses; no held-out test set was used and no generalizable predictive performance is claimed. The scripts used for model training, feature importance, and SHAP analysis are provided in File S1.

### Structural analysis and visualization

To identify the IBB domain and armadillo (ARM) repeats in importin α proteins, we used InterProScan via the European Molecular Biology Laboratory–European Bioinformatics Institute web server [36]. To annotate the HEAT repeat regions in importin β, we used the HHrepID server with default parameters [37–39].

Structural visualization and figure preparation were performed using UCSF ChimeraX (version 1.9, Resource for Biocomputing, Visualization, and Informatics, University of California, San Francisco) [40].

## Results

### Predicted Cap–IPO9 complexes show the lowest mean Δ*iG* across all tested circoviruses

All importins were ranked by mean Δ*iG* (the solvation free energy gain upon interface formation, as calculated by PDBePISA) of the predicted Cap–importin complexes across 11 viruses. IPO9 showed the lowest average Δ*iG* (−7.89 kcal/mol) among all importins tested (Fig. 1A and B).

**Fig. 1.**
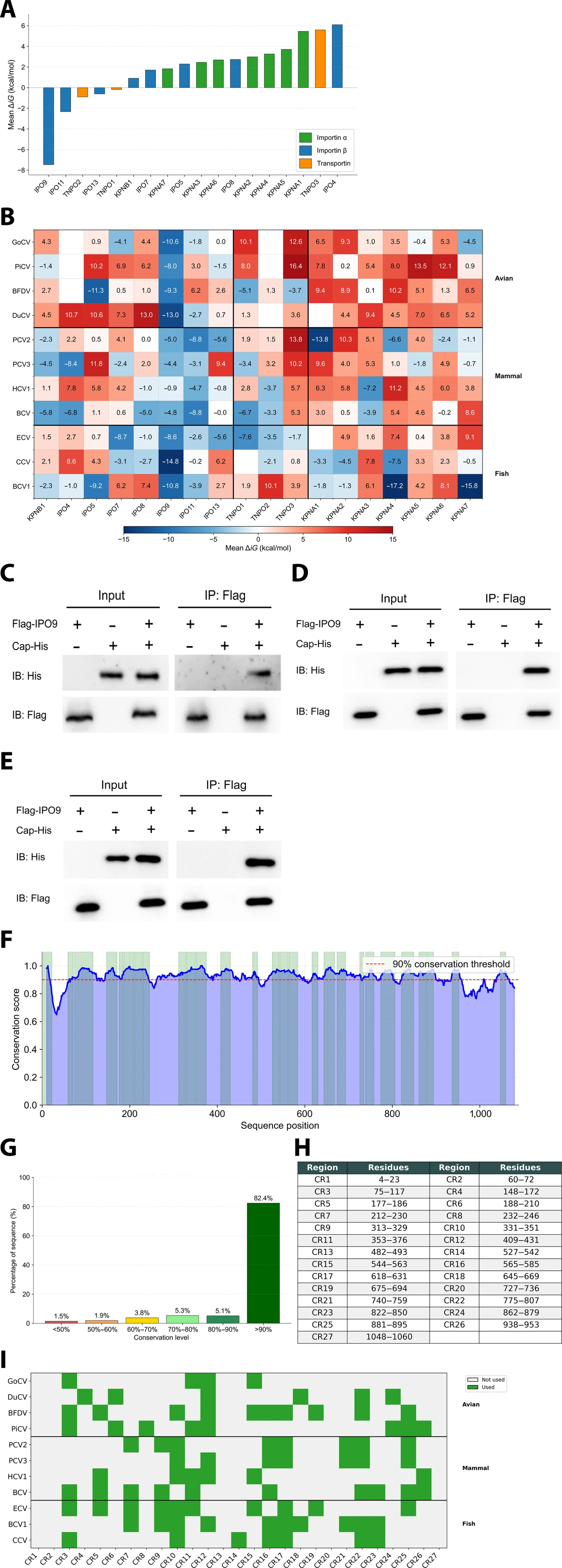
Analysis of capsid protein (Cap)–importin-9 (IPO9) binding and IPO9 conservation. (A) Mean Δ*iG* of each importin across all 11 circovirus species. Importins are ranked from lowest to highest Δ*iG*. (B) Heatmap of Δ*iG* for all circovirus–importin combinations. (C to E) Co-immunoprecipitation (Co-IP) validation of Cap–IPO9 interactions for goose circovirus (GoCV; avian), human circovirus 1 (HCV1; mammalian), and catfish circovirus (CCV; fish). (F) Conservation profile of IPO9 across 10 vertebrates. The conservation score (0 to 1) represents the frequency of the most common amino acid at each position. The red dashed line marks the 90% conservation threshold; green bars indicate conserved regions (CR1 to CR27) where the score is ≥0.9 for ≥10 consecutive residues. (G) Distribution of conservation levels across the IPO9 sequence. (H) Table of 27 conserved regions identified in IPO9. (I) Heatmap showing participation of conserved IPO9 regions in viral interactions.

IPO9 showed negative Δ*iG* with all 11 circovirus capsid proteins tested. Other members of the importin β group also showed a relatively high proportion of negative interactions, while members of the importin α group showed a much lower proportion (Fig. 1B).

Co-IP assays were performed for 3 representative virus–host pairs, one from each host class: GoCV (avian), HCV1 (mammalian), and CCV (fish). Co-IP assays detected specific physical interaction between Cap and IPO9 for all 3 pairs (Fig. 1C to E). Full-length immunoblots are provided in Fig. S10.

Multiple sequence alignment revealed that IPO9 is highly conserved, with an overall mean conservation score of 0.92 (Fig. 1G). The conservation profile showed that 82.4% of IPO9 residues are highly conserved (>90% identity), while 5.3% of residues show variation (<70% conservation) (Fig. 1H).

Twenty-seven conserved regions (CR1 to CR27) were identified with conservation scores exceeding 90% for at least 10 consecutive amino acids (Fig. 1I). These regions are distributed throughout the protein sequence (positions 4 to 1060).

After mapping the interface residues from each Cap–IPO9 complex onto the IPO9 sequence, we found that 22 of the 27 conserved regions were targeted by at least one virus (Fig. 1F). The most frequently contacted regions were CR10 (positions 331 to 351), CR12 (409 to 431), and CR22 (775 to 807), each engaged by 7 out of 11 viruses.

### Cap–importin β complexes exhibit the lowest mean Δ*iG* among the 3 importin groups analyzed

Comparison across the 3 importin groups analyzed here showed that the predicted Cap–importin β complexes had the lowest median Δ*iG*, regardless of host origin (Fig. 2A). The median Δ*iG* for Cap–importin β complexes was −0.43 kcal/mol, compared with 1.80 kcal/mol for transportin and 3.25 kcal/mol for importin α. The difference was more pronounced for importin β *vs.* importin α (*P* < 0.0001) than for importin β *vs.* transportin (*P* = 0.0410). After Bonferroni correction for 3 pairwise comparisons (adjusted threshold *P* = 0.0167), only the difference between importin β and importin α remained significant; the difference between importin β and transportin did not. Because multiple Cap–importin combinations share the same Cap sequence or the same importin, these *P* values should be interpreted as exploratory. An importin-level sensitivity analysis is provided in Fig. S11.

**Fig. 2.**
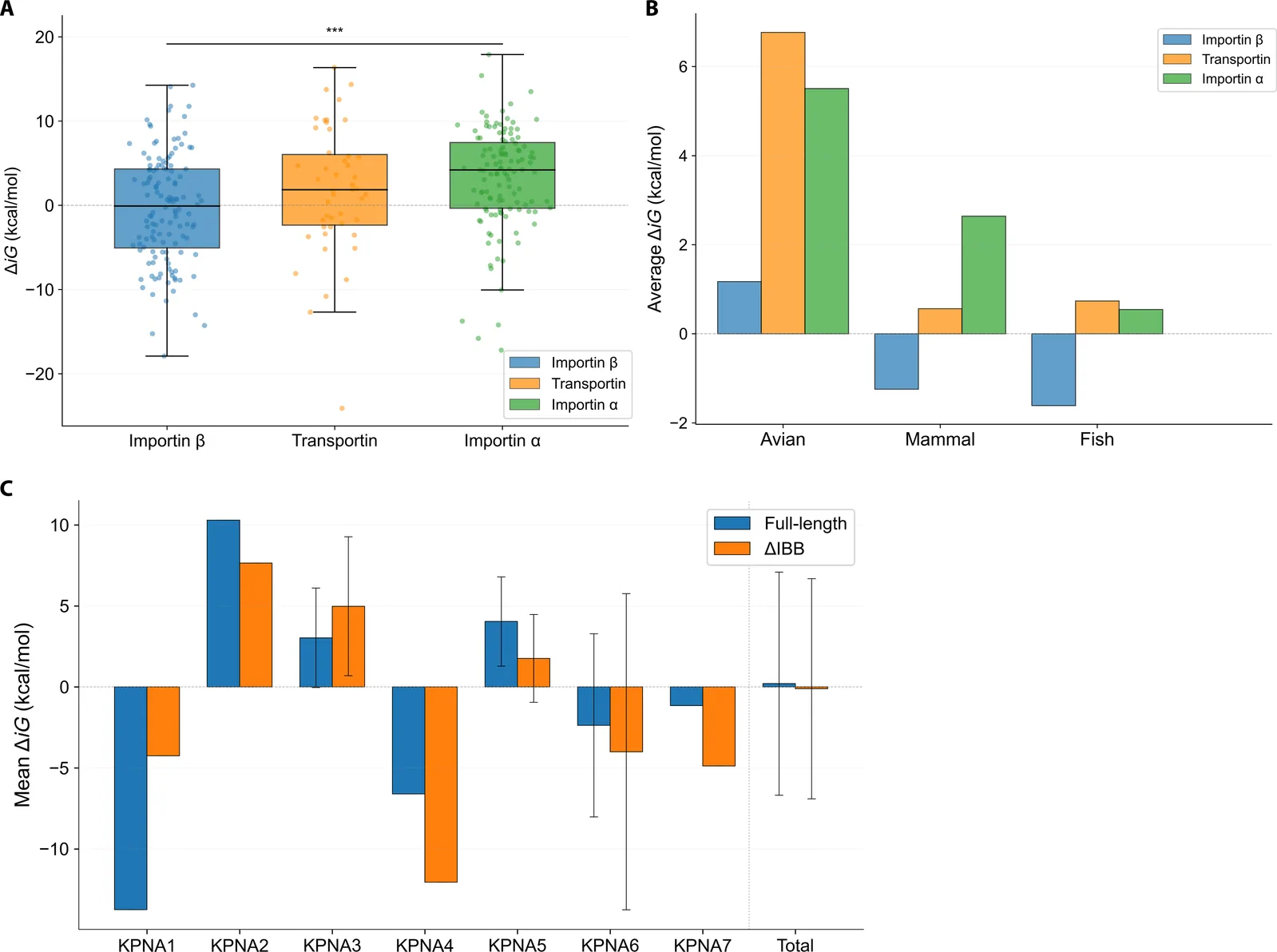
Comparison of Δ*iG* across the 3 importin groups. (A) Δ*iG* distribution by importin group. Boxes show median and interquartile ranges; whiskers extend to 1.5× the interquartile range (***P < 0.001, Mann–Whitney *U* test). (B) Mean Δ*iG* by host class and importin group. Capsid protein (Cap)–importin β complexes show the lowest mean Δ*iG* in all 3 host classes. Error bars and significance markers are omitted for clarity; statistical comparisons are reported in the main text. (C) Comparison of Δ*iG* values between full-length and ΔIBB importin α constructs for porcine circovirus type 2 (PCV2) Cap complexes. IBB, importin β-binding.

A similar pattern was observed across all 3 host classes included in this study when examining mean Δ*iG* by host class and importin group (Fig. 2B). In avian circoviruses, the lowest mean Δ*iG* was observed for Cap–importin β complexes (1.31 ± 7.09 kcal/mol), compared with transportin (5.69 ± 7.08 kcal/mol, *P* = 0.0918) and importin α (5.51 ± 4.70 kcal/mol, *P* = 0.0045). In mammalian circoviruses, Cap–importin β complexes also showed a lower mean Δ*iG* (−1.25 ± 5.27 kcal/mol) than transportin (0.56 ± 7.90 kcal/mol, *P* = 0.0971) and importin α (2.64 ± 5.69 kcal/mol, *P* = 0.0003). Among the 3 host classes, the lowest mean Δ*iG* was observed for Cap–importin β complexes from fish circoviruses (−1.61 ± 6.88 kcal/mol). Within fish circoviruses, Cap–importin β complexes also had the lowest mean Δ*iG*, followed by importin α (0.54 ± 7.67 kcal/mol) and transportin (0.74 ± 7.10 kcal/mol). ΔIBB importin α constructs did not show systematically lower Δ*iG* values than full-length constructs (overall means: ΔIBB = −0.11 kcal/mol; full-length = 0.20 kcal/mol; Fig. 2C).

### Host-class-associated differences in Cap–importin Δ*iG*

#### Avian circoviruses show a higher overall Δ*iG* than mammalian and fish circoviruses

Avian circoviruses showed a higher overall Δ*iG* than mammalian and fish circoviruses, with a median Δ*iG* of 4.40 kcal/mol for avian compared to 0.75 kcal/mol for both mammalian and fish (Fig. 3A, *P* = 0.0003 for avian *vs.* mammals, *P* = 0.0005 for avian *vs.* fish, Mann–Whitney *U* test). Because multiple Cap–importin combinations share the same Cap sequence or the same importin, these *P* values should be interpreted as exploratory.

**Fig. 3.**
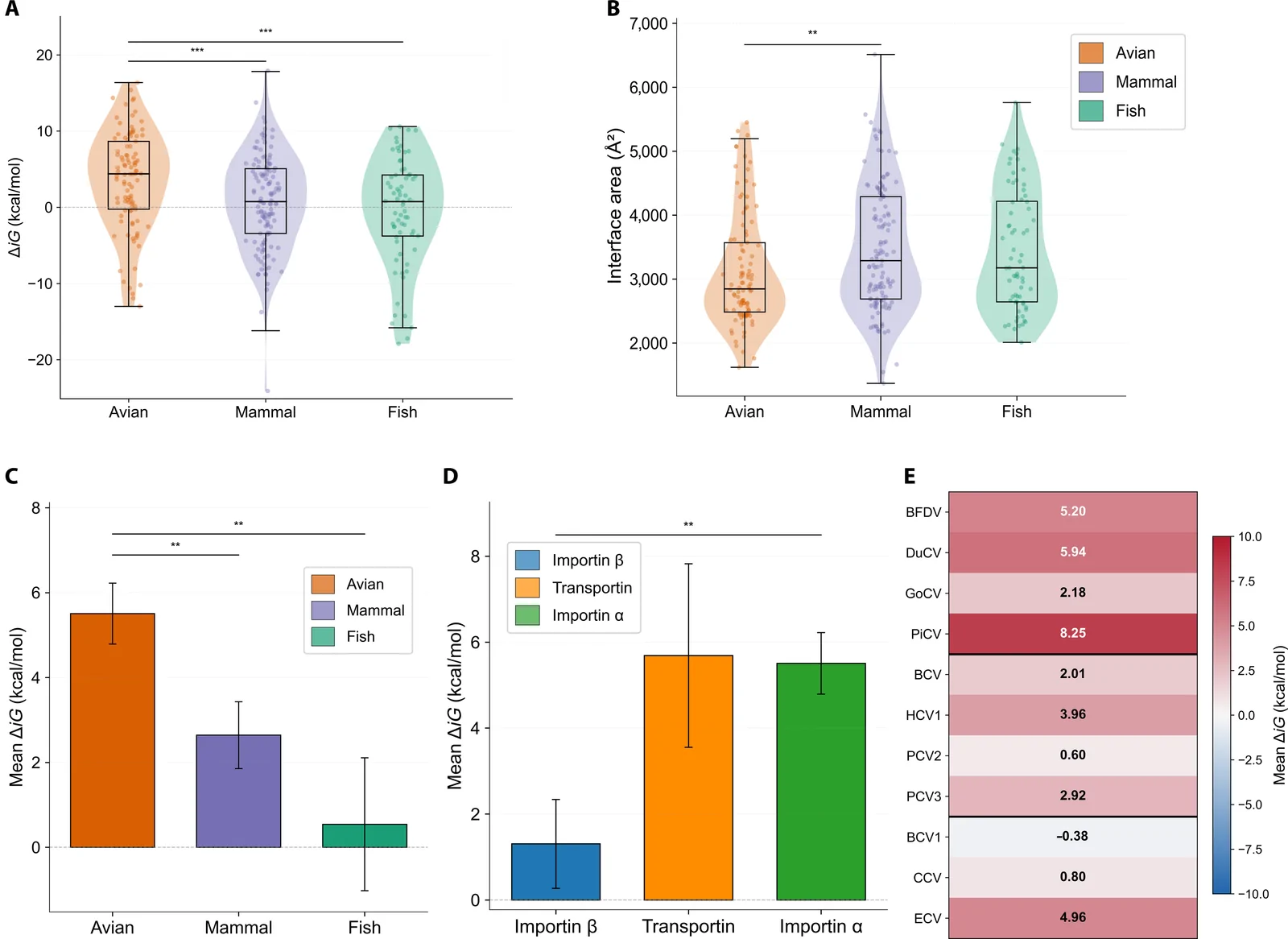
Predicted capsid protein (Cap)–importin Δ*iG* patterns of avian circoviruses. (A) Δ*iG* distribution by host class. (B) Interface area distribution by host class. (C) Mean Δ*iG* for Cap–importin α complexes by host class. (D) Mean Δ*iG* for avian circovirus Cap–importin complexes across the 3 importin groups (**P < 0.01; ***P < 0.001, Mann–Whitney *U* test). *P* values are exploratory and were not corrected for multiple comparisons. (E) Heatmap of importin α Δ*iG* for Cap–importin α complexes across individual circovirus species. Viruses are grouped by host origin.

The interface areas of avian circovirus complexes were smaller than those of mammalian or fish circoviruses (Fig. 3B). Avian complexes had a median interface area of 2,846.8 Å^2^, compared to 3,288.4 Å^2^ for mammalian and 3,176.1 Å^2^ for fish complexes (*P* = 0.0032 for avian *vs.* mammals; *P* = 0.0258 for avian *vs.* fish, Mann–Whitney *U* test). Mammalian and fish circoviruses showed comparable interface areas.

Avian circoviruses showed a higher mean Δ*iG* for complexes with importin α than mammalian and fish circoviruses (Fig. 3C). The mean Δ*iG* for avian circoviruses was 5.51 ± 4.70 kcal/mol, compared to 2.64 ± 5.69 kcal/mol for mammalian and 0.54 ± 7.67 kcal/mol for fish circoviruses (*P* = 0.0124 for avian *vs.* mammals, *P* = 0.0069 for avian *vs.* fish, Mann–Whitney *U* test). Mammalian and fish circoviruses showed comparable mean Δ*iG* for importin α complexes.

Within avian circoviruses, Cap–importin β complexes had a lower mean Δ*iG* (1.31 ± 7.09 kcal/mol) than Cap–importin α complexes (5.51 ± 4.70 kcal/mol, *P* = 0.0045), while no significant difference was observed between importin β and transportin (mean 5.69 ± 7.08 kcal/mol, *P* = 0.0918) (Fig. 3D). Virus-level sensitivity analyses for Fig. 3A to C and an importin-level sensitivity analysis for Fig. 3D are provided in Fig. S12A to D. The heatmap of Δ*iG* for Cap–importin α complexes showed that avian circoviruses had positive values, with PiCV showing the highest mean Δ*iG* (8.25 ± 6.08 kcal/mol) (Fig. 3E).

#### IPO11 shows the lowest mean Δ*iG* among importins in mammalian circoviruses

In mammalian circoviruses, IPO11 was the top-ranked importin by mean Δ*iG* (−6.01 ± 1.24 kcal/mol), followed by IPO9 (−3.15 ± 1.41 kcal/mol) (Fig. 4A).

**Fig. 4.**
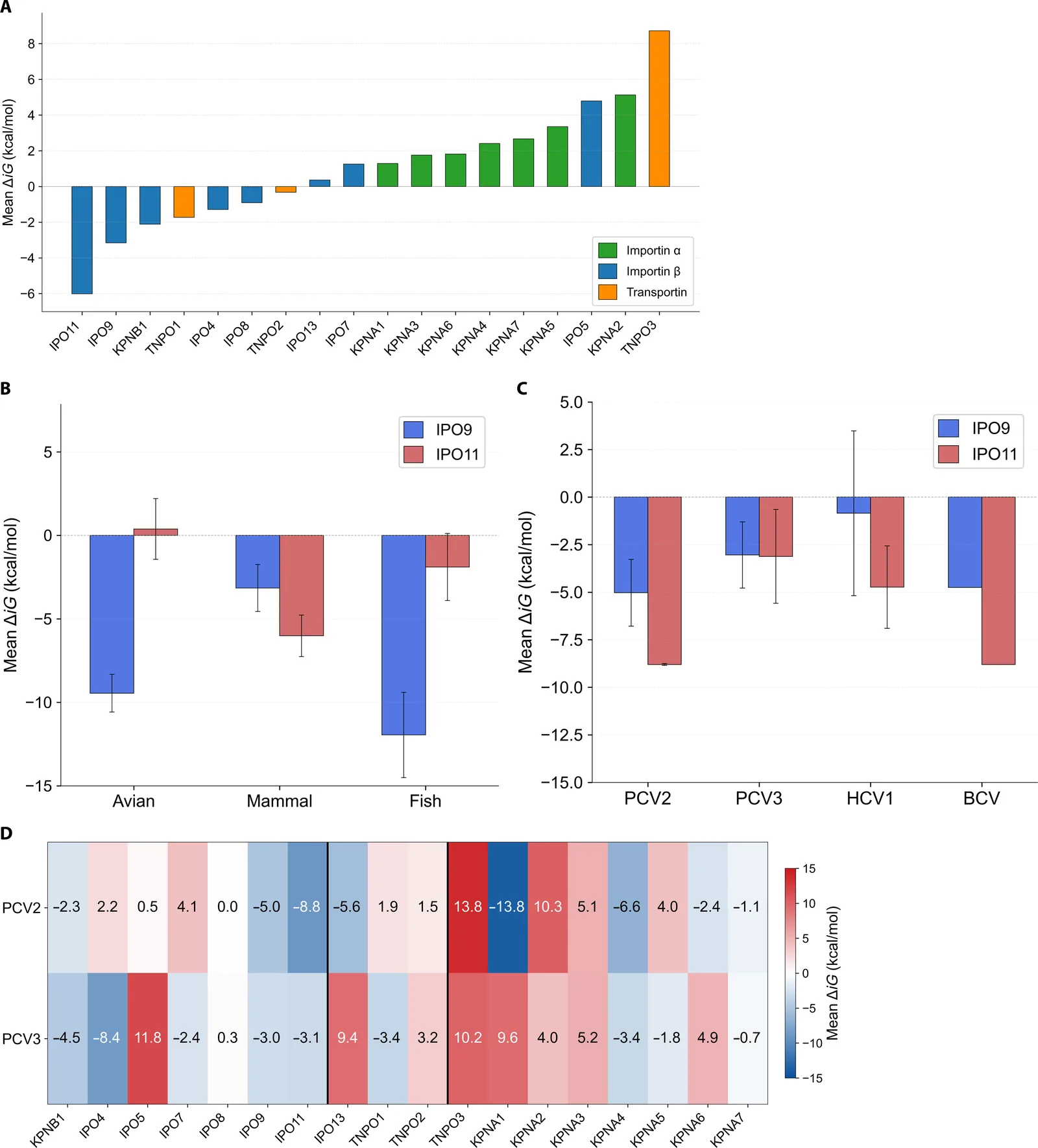
Predicted capsid protein (Cap)–importin Δ*iG* profiles of mammalian circovirus capsids. (A) Ranking of importins by mean Δ*iG* in mammalian circoviruses. (B) Cross-species comparison of importin-9 (IPO9) and IPO11 Δ*iG* by host class. (C) IPO9 and IPO11 Δ*iG* for individual mammalian circoviruses (porcine circovirus type 2 [PCV2], PCV3, human circovirus 1 [HCV1], and bat circovirus [BCV]). (D) Heatmap comparing PCV2 and PCV3 Δ*iG* across all 18 importins. Standard deviations are omitted where only one unique Cap–importin combination is available.

A cross-species comparison suggested that within the sampled dataset, the lower mean Δ*iG* for IPO11 relative to that for IPO9 was limited to mammalian hosts; in avian and fish circoviruses, IPO9 showed a lower mean Δ*iG* than IPO11 (Fig. 4B). In PCV2, HCV1, and BCV, IPO11 showed lower Δ*iG* values than IPO9, while in PCV3, IPO9 and IPO11 showed comparable Δ*iG* (Fig. 4C).

Despite sharing the same host (swine), PCV2 and PCV3 displayed distinct Δ*iG* profiles (Fig. 4D). For PCV2, the lowest mean Δ*iG* values were observed for KPNA1 (mean −13.75 kcal/mol), IPO11 (−8.80 ± 0.07 kcal/mol), IPO13 (−5.55 kcal/mol), and IPO9 (−5.03 ± 3.05 kcal/mol). For PCV3, the lowest mean Δ*iG* values were observed for IPO4 (−8.45 kcal/mol), KPNB1 (−4.55 kcal/mol), and IPO11 (−3.12 kcal/mol). The largest differences between the 2 viruses were observed for KPNA1 (PCV2 lower by 23.30 kcal/mol), IPO13 (PCV2 lower by 14.55 kcal/mol), and IPO5 (PCV2 lower by 11.30 kcal/mol).

#### Fish circovirus Cap–importin complexes show a higher proportion and a broader range of low-Δ*iG* interactions

Fish circoviruses showed the highest proportion of interactions with Δ*iG* < −10 kcal/mol among all host classes across the tested thresholds (−8, −10, and −12 kcal/mol) (Fig. 5A). At Δ*iG* < −10 kcal/mol, 7 of 68 fish circovirus Cap–importin pairs (10.3%) fell below the threshold, compared to 5 of 101 (5.0%) for avian and 4 of 129 (3.1%) for mammalian circoviruses. The same pattern was observed at −8 and −12 kcal/mol.

**Fig. 5.**
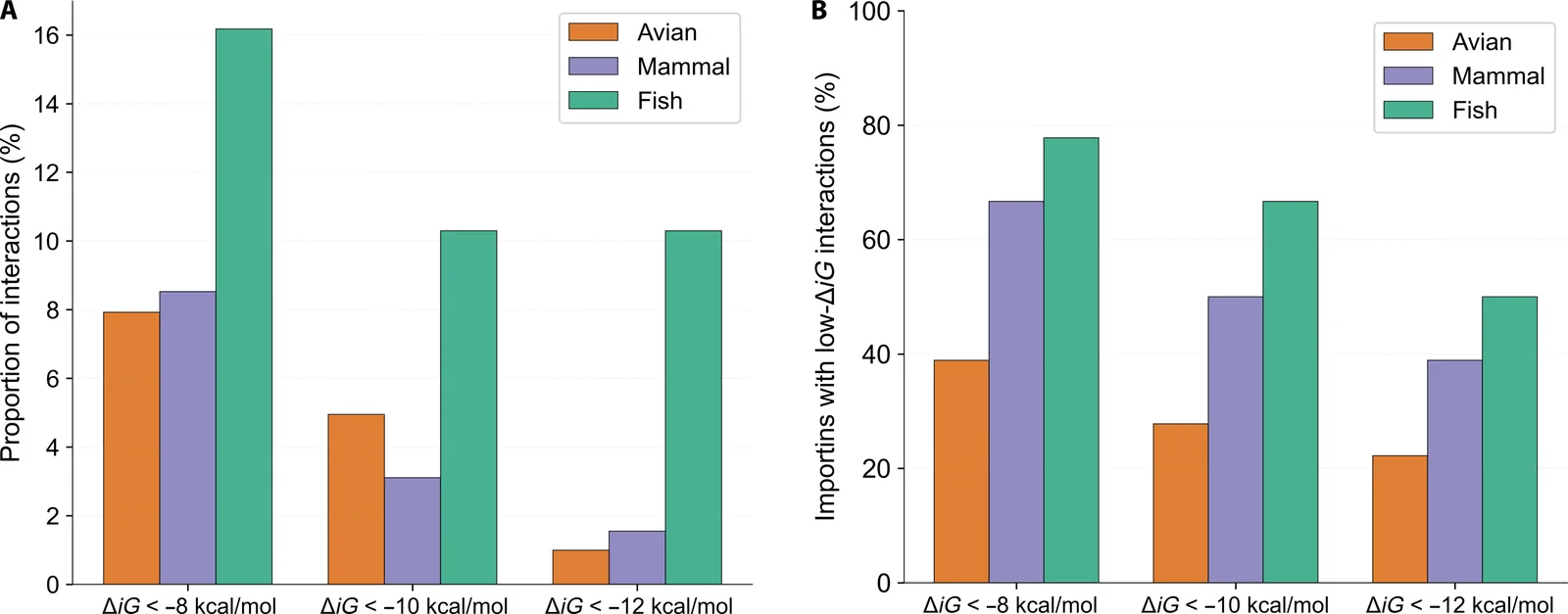
Fish circovirus models show a higher proportion and a broader range of importins with low-Δ*iG* interactions. (A) Proportion of unique capsid protein (Cap)–importin pairs with Δ*iG* below the indicated thresholds (−8, −10, and −12 kcal/mol), calculated within each host class. (B) Percentage of the 18 importins with at least one conformation below the indicated threshold within each host class. Panel (A) uses the pair-averaged Δ*iG* criterion, whereas panel (B) uses the presence of at least one low-Δ*iG* conformation per importin.

Fish circoviruses also showed a wider range of importins with low-Δ*iG* interactions. For each host class, we counted the number of importins for which at least one conformation had a Δ*iG* below the threshold. At Δ*iG* < −10 kcal/mol, fish circoviruses had 12 of 18 importins (66.7%) with at least one low-Δ*iG* conformation, compared to 9 (50.0%) for mammalian and 5 (27.8%) for avian circoviruses (Fig. 5B). The same ranking was observed at −8 and −12 kcal/mol (Fig. 5B). The −10 kcal/mol threshold corresponds approximately to the 5th percentile of the overall Δ*iG* distribution (Fig. S13) and −8 and −12 kcal/mol to the 10th and 3rd percentiles, respectively.

### NLS interface features differ across host classes

NLS regions were predicted for all 11 circovirus Cap proteins (Table S4). The predicted NLS length ranged from 27 to 47 residues and accounted for 10.7% to 20.3% of the Cap sequence. The mean NLS bond contribution ranged from 48.4% (ECV) to 75.0% (DuCV) across viruses, with an overall mean of 61.6% (Table 1). For the 4 viruses with experimentally defined NLS regions, the predicted regions overlapped with the experimental ranges: DuCV (2 to 36 *vs.* 1 to 36), BFDV (15 to 41 *vs.* 16 to 56), PCV2 (4 to 41 *vs.* 1 to 41), and PCV3 (2 to 33 *vs.* 1 to 33).

**Table 1.** Predicted NLS regions in circovirus Cap proteins

Virus	Predicted NLS region	NLS length (amino acids)	Cap length (amino acids)	Mean NLS bond contribution (%)
DuCV	R2–R36 (experimental 1–36 [72])	35	257	75.0
GoCV	R5–K38	34	250	62.9
PiCV	R2–K48	47	273	64.6
BFDV	R15–R41 (experimental 16–56 [23])	27	247	52.8
PCV2	P4–K41 (experimental 1–41 [20])	38	233	63.9
PCV3	R2–R33 (experimental 1–33 [73])	32	255	65.9
HCV1	R7–R33	27	214	62.5
BCV	R4–K42	39	235	66.2
ECV	D8–N39	32	255	48.4
BCV1	R12–R40	29	257	52.4
CCV	R4–R35	32	227	55.4

Cap, capsid protein; NLS, nuclear localization signal; DuCV, duck circovirus; GoCV, goose circovirus; PiCV, pigeon circovirus; BFDV, beak and feather disease virus; PCV2 and PCV3, porcine circovirus types 2 and 3; HCV1, human circovirus 1; BCV, bat circovirus; ECV, eel circovirus; BCV1, bream circovirus 1; CCV, catfish circovirus

The number of predicted NLS residues present at each modeled interface is summarized in Table 2. Mean interface NLS residues ranged from 18.4 (BFDV) to 29.1 (PCV2) across viruses. The per-interface minimum ranged from 0 (CCV) to 17 (PCV2), and the per-interface maximum ranged from 26 (BFDV) to 42 (PiCV). The ratio of mean interface NLS residues to NLS length ranged from 58.6% (PiCV) to 80.0% (BCV1).

**Table 2.** Predicted NLS residues at modeled Cap–importin interfaces by virus

Virus	Mean NLS residues on interface (amino acids)	Minimum NLS residues on interface (amino acids)	Maximum NLS residues on interface (amino acids)	Mean NLS residues on interface/NLS length
DuCV	25.54	13	33	72.97%
GoCV	24.99	15	34	73.50%
PiCV	27.54	9	42	58.60%
BFDV	18.38	5	26	68.07%
PCV2	29.10	17	38	76.58%
PCV3	25.44	12	32	79.50%
HCV1	20.88	9	29	77.33%
BCV	27.38	10	39	70.21%
ECV	22.57	1	32	70.53%
BCV1	23.22	8	29	80.01%
CCV	21.88	0	32	68.38%

NLS, nuclear localization signal; Cap, capsid protein; DuCV, duck circovirus; GoCV, goose circovirus; PiCV, pigeon circovirus; BFDV, beak and feather disease virus; PCV2 and PCV3, porcine circovirus types 2 and 3; HCV1, human circovirus 1; BCV, bat circovirus; ECV, eel circovirus; BCV1, bream circovirus 1; CCV, catfish circovirus

Within-virus and between-virus variability were also compared for 5 NLS interface features (Table 3). Within-virus variability exceeded between-virus variability for NLS interface occupancy (SD ratio = 2.11), NLS bond contribution (1.96), and NLS interface residues (1.54). NLS net charge showed comparable variability (ratio = 0.98), and NLS basic residue ratio showed lower within-virus variability (ratio = 0.40).

**Table 3.** Within-virus and between-virus variability of NLS interface features

Feature	Within-virus SD	Between-virus SD	Ratio
NLS interface occupancy	0.1453	0.0688	2.11
NLS basic residue ratio	0.0424	0.1068	0.40
NLS net charge	2.9729	3.0314	0.98
NLS bond contribution	0.1528	0.0778	1.96
NLS interface residues	4.8439	3.1381	1.54

NLS, nuclear localization signal

Three NLS interface features—interface occupancy, basic residue ratio, and net charge—were compared across avian, mammalian, and fish circoviruses (Fig. 6A to C). NLS interface occupancy was highest in mammalian circoviruses (0.777 ± 0.141), followed by fish (0.747 ± 0.184) and avian circoviruses (0.673 ± 0.141) (Fig. 6A). NLS basic residue ratio was highest in avian circoviruses (0.656 ± 0.062), followed by mammalian (0.614 ± 0.083) and fish circoviruses (0.544 ± 0.146) (Fig. 6B). NLS net charge was highest in mammalian circoviruses (16.0 ± 3.4), followed by avian (15.6 ± 3.5) and fish circoviruses (12.4 ± 4.6) (Fig. 6C).

**Fig. 6.**
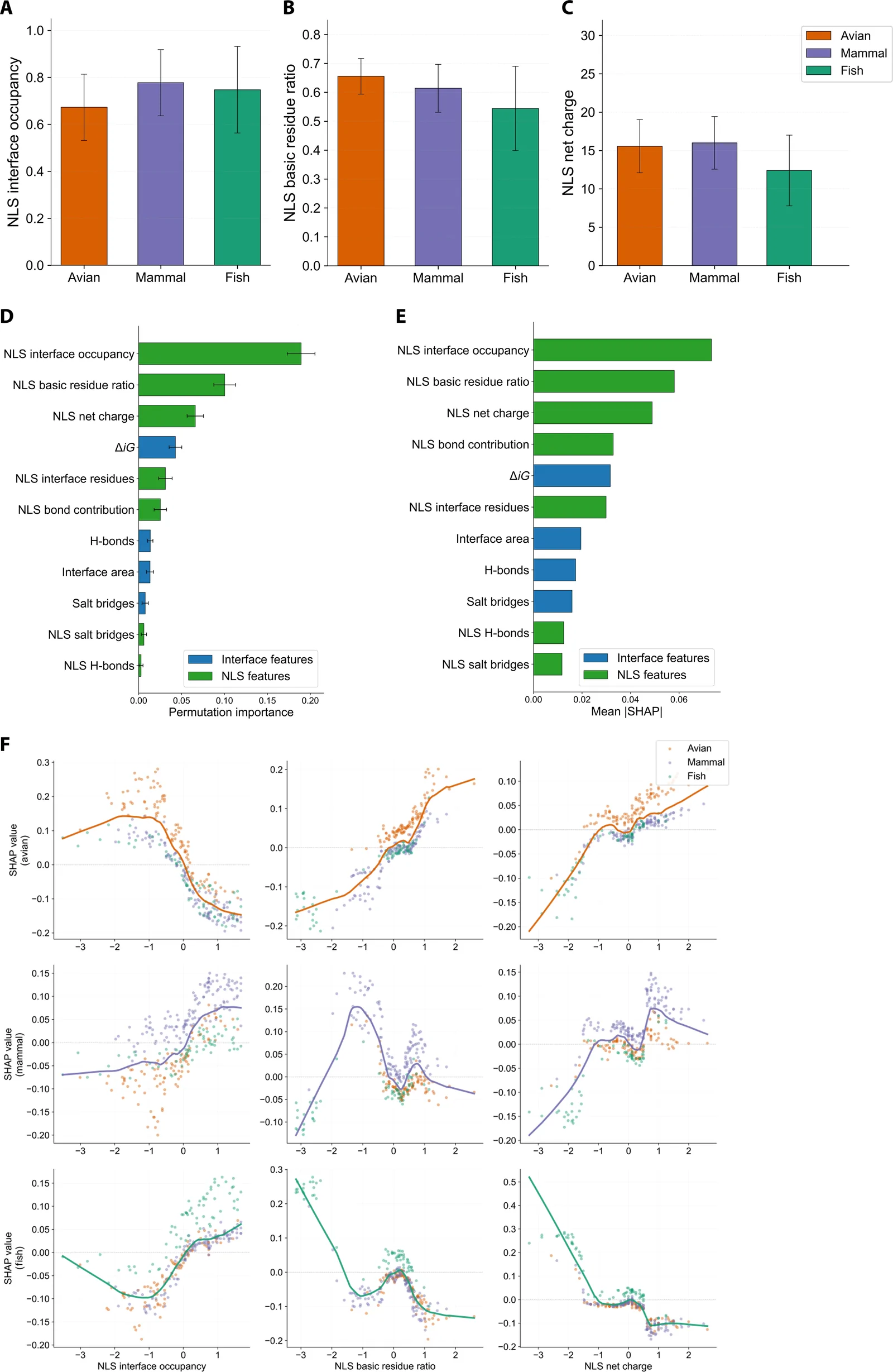
Nuclear localization signal (NLS) interface features across host classes. (A) NLS interface occupancy. (B) NLS basic residue ratio. (C) NLS net charge. Values are mean ± SD across unique capsid protein (Cap)–importin combinations. (D) Permutation importance (30 permutations). (E) Shapley additive explanations (SHAP) importance. NLS features are shown in green; interface features, in blue. (F) SHAP dependence plots for 3 NLS features. Rows correspond to the target class (avian, mammal, and fish), and columns correspond to NLS interface occupancy, NLS basic residue ratio, and NLS net charge.

Since multiple Cap–importin combinations share the same Cap sequence, these observations are not fully independent, and formal significance testing was not performed. Virus-level values showed the same direction of differences for all 3 features (Fig. S14).

Permutation importance and SHAP values both identified NLS interface occupancy, NLS basic residue ratio, and NLS net charge as the top 3 features for host-class discrimination (Fig. 6D and E). SHAP dependence plots for the top 3 NLS features are shown in Fig. 6F. The relationships between NLS features and host-class discrimination were nonlinear and varied across classes.

### Structural features of predicted NLS residues in Cap–importin complexes

To examine whether the host-associated differences in NLS interface features correspond to differences in structural binding modes, we selected low-Δ*iG* models across the 3 host classes for 3 importin groups: KPNA6 (importin α), KPNB1 (importin β), and TNPO1 (transportin). For each importin, we selected one model from each host class—avian, mammalian, and fish—prioritizing the lowest Δ*iG* within each class (Data File S1). In total, 9 models were examined (Fig. 7A). Confidence metrics for the models shown in Fig. 7 are provided in Table S5.

**Fig. 7.**
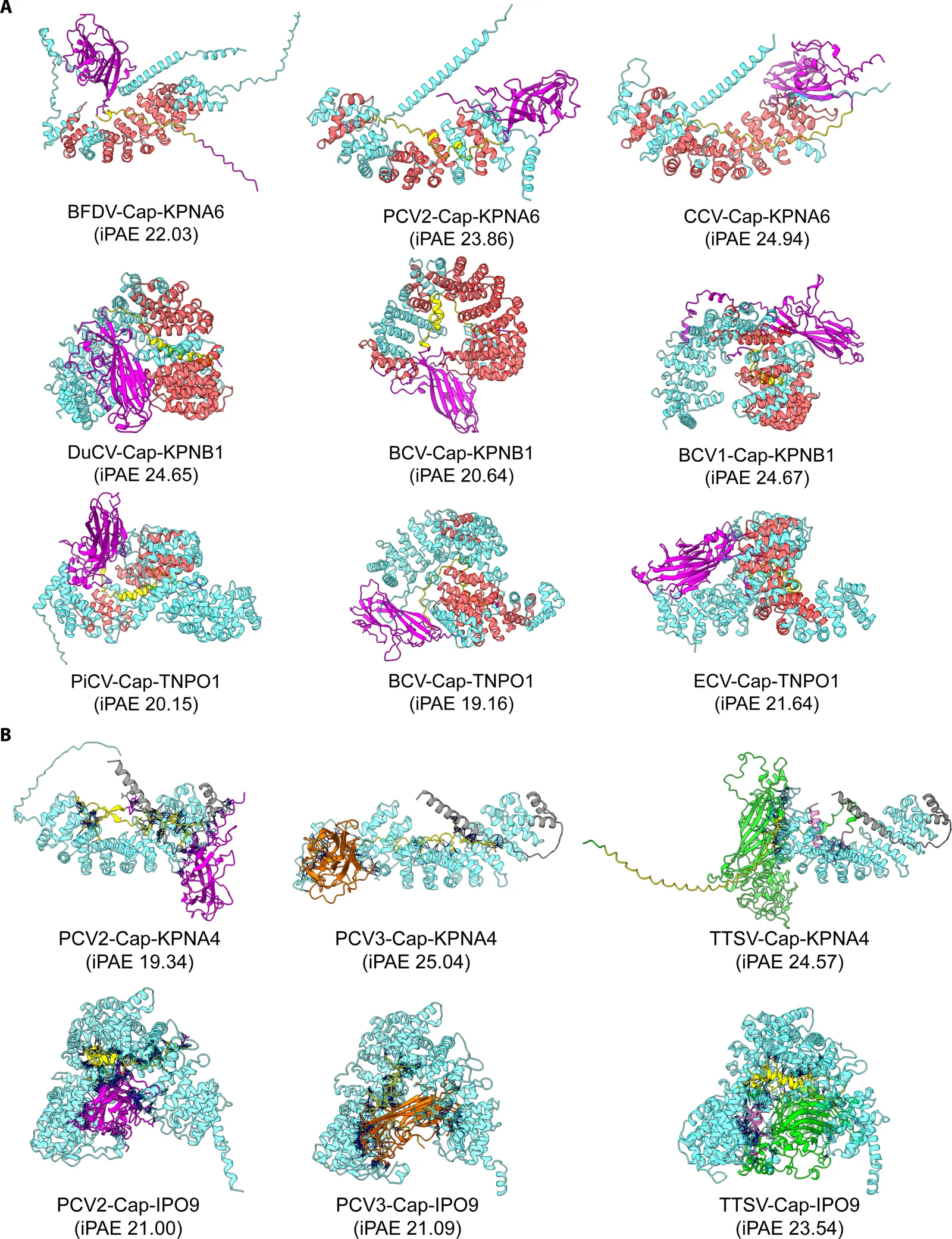
Structural comparison of predicted nuclear localization signal (NLS) residues at capsid protein (Cap)–importin interfaces across circovirus and anellovirus capsids. (A) Representative circovirus Cap–importin models across 3 host classes for KPNA6, KPNB1, and TNPO1. Importin is shown in cyan, capsid protein in magenta, NLS region in yellow, and ARM or HEAT repeats involved in contacts in red. (B) Comparison of circovirus (porcine circovirus type 2 [PCV2] and PCV3) and anellovirus (torque teno sus virus [TTSV]) capsid binding modes with KPNA4 and importin-9 (IPO9). Importin is shown in cyan, importin β-binding (IBB) domain in gray, PCV2 capsid in magenta, PCV3 capsid in orange, TTSV capsid in green, N-terminal NLS in yellow, and C-terminal NLS in pink. Contacts between Cap and importin are indicated by dark blue dashed lines. Confidence metrics and interface predicted aligned error (iPAE) values for the displayed model are provided in Table S5.

In the Cap–KPNA6 models, the NLS regions of all 3 viruses bound within the ARM repeat domains of importin α. For BFDV, the NLS engaged ARM repeats 1 through 8, with the most extensive contacts in ARM repeats 1, 3, and 4. For PCV2, the contacts were more dispersed, involving ARM repeats 1, 4, 5, 6, and 8, with the majority of interactions concentrated in ARM repeats 5 and 6. For CCV, the NLS showed the broadest distribution, contacting ARM repeats 1, 3, 4, 5, 6, 7, 8, and 9, with ARM repeats 5 and 6 showing the highest contact density. Detailed contact maps for all KPNA6 models are provided in Tables S6 to S8.

For Cap-KPNB1, the binding patterns differed more markedly. DuCV Cap engaged HEAT repeats 10 to 18, with contacts distributed across the C-terminal half of KPNB1. BCV showed the broadest distribution, with contacts spanning HEAT repeats 1 to 15, involving both N-terminal and central regions. BCV1 showed a more restricted pattern, with contacts primarily in HEAT repeats 7, 10, 12, and 14 to 17. The detailed contact maps for all KPNB1 models are provided in Tables S9 to S11.

In the Cap–TNPO1 models, the NLS regions of the 3 viruses bound to overlapping but distinct regions of the HEAT repeat domain. For PiCV Cap–TNPO1, the NLS engaged multiple HEAT repeats from 4 through 12, with contacts concentrated in HEAT repeats 7 to 9 and 12. For BCV, the NLS bound primarily to HEAT repeats 11 to A18, with extensive contacts in 13 and 14. For ECV, the NLS engaged HEAT repeats 7 to A16, with the most extensive contacts in 11 to 14. Detailed contact maps for Cap–TNPO1 models are provided in Tables S12 to S14.

We extended the same structural analysis to a representative anellovirus capsid protein from porcine TTSV, using PCV2 and PCV3 as circovirus references, with KPNA4 and IPO9 as representative importins (Fig. 7B). In the TTSV models, the N-terminal NLS, although longer and containing a higher density of basic residues, was not the primary binding interface of many predicted models (Fig. S15C and D).

In the Cap–KPNA4 models, TTSV Cap showed limited contacts with KPNA4, with the C-terminal NLS appearing obstructed by the IBB domain. PCV3 Cap showed a similar but less severe obstruction, forming noticeably more contacts with KPNA4 than TTSV. PCV2 Cap displayed the most extensive interactions, with its NLS threading between the IBB domain and the first 2 ARM repeats. In Cap–IPO9, the C-terminal NLS and the N-terminal NLS of TTSV both participated in the binding interface. In TTSV, the C-terminal NLS, rather than the N-terminal NLS, appeared to be the primary binding interface across multiple importins.

## Discussion

### IPO9 as a candidate broadly interacting importin among the circoviruses examined—Structural basis, evolutionary conservation, and biological implications

IPO9 is a member of the importin β family that mediates nuclear import through direct cargo recognition without requiring importin α as an adaptor.

Across all tested virus–host combinations, Cap–IPO9 complexes consistently showed lower Δ*iG* values than other Cap–importin complexes. To test whether these predicted interactions could be detected at the cellular level, we performed Co-IP assays for 3 representative Cap–IPO9 pairs, one from each host class: GoCV (avian), HCV1 (mammalian), and CCV (fish). All 3 pairs showed specific physical interaction between Cap and IPO9 (Fig. 1C to E). These results support the predicted Cap–IPO9 interactions and indicate that the interactions are not artifacts of computational modeling alone.

Structural models were generated for each circovirus Cap protein in complex with IPO9 (Fig. 8). These models indicated that although the capsid proteins differ in sequence, they all target similar surfaces on IPO9, mainly the HEAT repeat regions involved in cargo recognition.

**Fig. 8.**
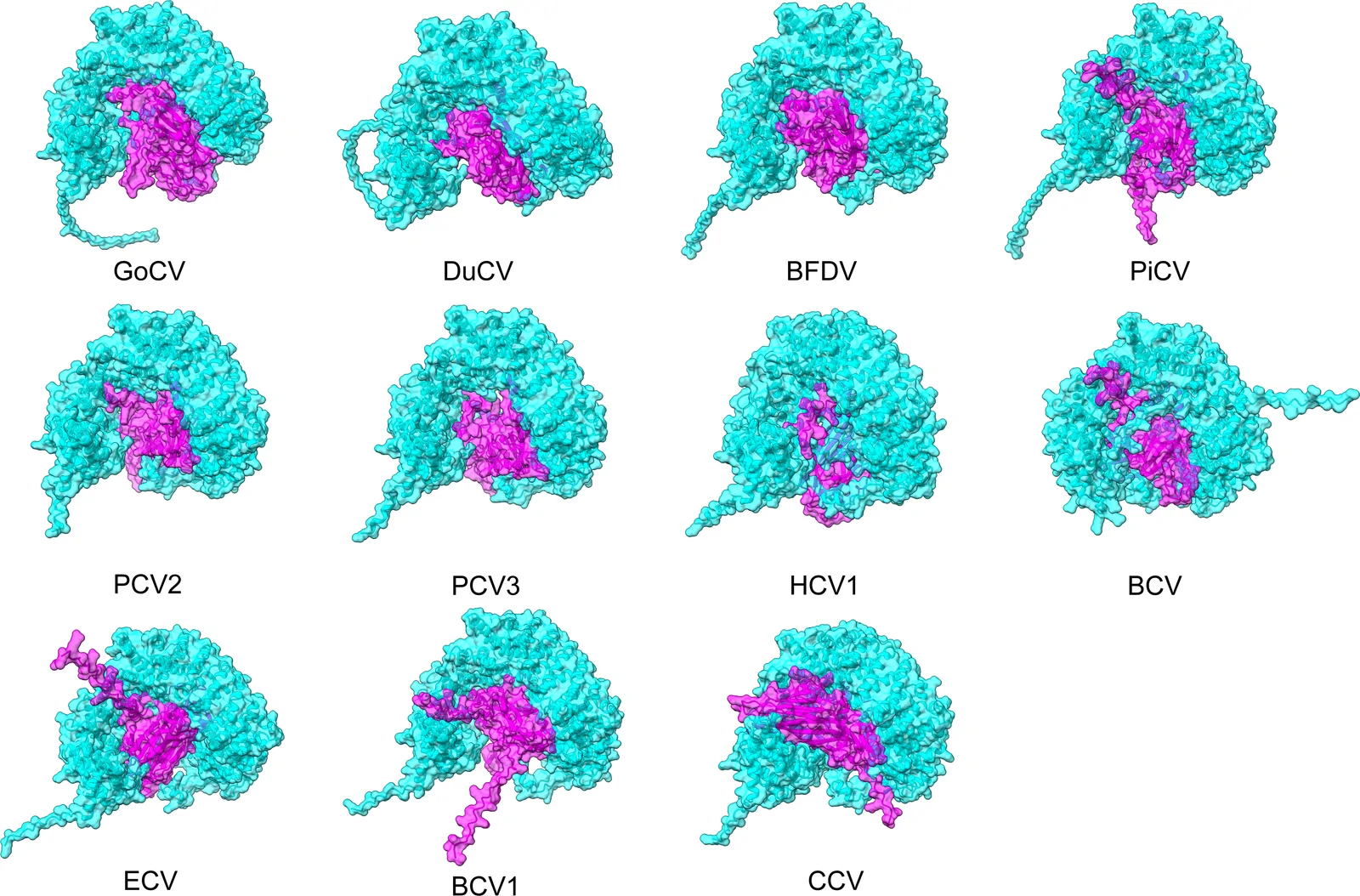
Structural overview of circovirus capsid protein (Cap)–importin-9 (IPO9) complexes. Cartoon representations of IPO9 (cyan) in complex with capsid proteins (magenta) from all 11 circovirus species are displayed. Viruses are ordered by host class: avian (goose circovirus [GoCV], duck circovirus [DuCV], beak and feather disease virus [BFDV], and pigeon circovirus [PiCV]), mammalian (porcine circovirus type 2 [PCV2], PCV3, human circovirus 1 [HCV1], and bat circovirus [BCV]), and fish (eel circovirus [ECV], bream circovirus 1 [BCV1], and catfish circovirus [CCV]). Despite sequence divergence, the predicted capsid proteins engage similar binding surfaces on IPO9.

IPO9 is composed of 20 antiparallel α-helical HEAT repeat pairs, which form a hollow, spiral-shaped scaffold. Four major flexible loops—the H7, H8, H18–19, and H19 loops—extend from this scaffold toward the interior cavity, constituting the core interface for cargo recognition. IPO9 is able to recognize specific 3-dimensional folds of its cargo proteins [41]. Its cargo repertoire includes structurally and functionally diverse proteins such as histones H2A–H2B, the transcription factor TFIIB (transcription factor II B), actin, and the proteasome [42]. For example, E26 transformation-specific (ETS) family transcription factors are recognized through their winged-helix DNA-binding domain. Cryo-electron microscopy structures reveal that IPO9 wraps around the entire ETS domain like a molecular “glove”, engaging multiple surface features throughout the fold [43]. A similar “glove-like” binding mode is observed in our AF3-predicted models of circovirus Cap–IPO9 complexes, where the interaction extends beyond the NLS region (Fig. S9). In contrast, in the TTSV Cap–IPO9 model (Fig. 7B), the larger capsid structure prevents TTSV from being similarly “wrapped” by IPO9. Although current prediction tools cannot precisely map every HEAT repeat on IPO9, the Cap–IPO9 models consistently display a more uniform binding mode compared to the other Cap–importin complexes shown in Fig. 7A.

IPO9 is highly conserved across the 10 host species examined in this study. This conservation is not restricted to these species; IPO9 also exhibits functional conservation across a wide range of organisms, from invertebrates to vertebrates [41]. It displays features characteristic of housekeeping genes, with broad expression across tissues and consistent presence during embryonic development [44]. IPO9 is responsible for transporting core histones, TATA-box-binding protein, TFIIB, and ribosomal proteins [45]. These are fundamental components for the assembly of chromatin and the transcriptional apparatus, which are indispensable in all eukaryotic cells.

IPO9 may be relevant to a broader range of viruses beyond circoviruses. It has been identified as a host factor required by flaviviruses [46], and it has also been implicated in HIV-1 (human immunodeficiency virus type 1) infection [47]. The fact that both RNA and DNA viruses appear to engage IPO9 makes it a potentially attractive target for viral exploitation.

Circoviruses possess extremely compact genomes and rely entirely on the host cell machinery for replication. Given the low mutation rate of DNA viruses and the constraints imposed by a minimalist genome structure, most random mutations are expected to be deleterious. Consequently, unlike RNA viruses such as influenza that frequently jump between hosts through rapid adaptive evolution, the broad host range of circoviruses is more likely the result of long term coevolution [48]. An accidental introduction into a new host species may be followed by an extended period of coexistence, during which rare beneficial mutations are gradually selected, leading to host adaptation [49].

However, to determine whether IPO9 actually plays a role in Cap nuclear import, future studies incorporating transcriptomic data—particularly the expression profiles of various importins in relevant cell types and tissues across different host species—will be required. Such analyses would help establish whether IPO9 has a uniquely important function beyond what is suggested by the structural models presented here.

### Energetic and structural basis for importin family selectivity of circovirus capsids

In this study, the Δ*iG* values of importin β-Cap complexes were lower than those of importin α-Cap complexes across all tested combinations. The IBB domain of importin α has a unique autoinhibitory mechanism and naturally occludes the cargo-binding region of importin α [50]. However, ΔIBB importin α constructs did not show systematically lower Δ*iG* values than full-length constructs, indicating that the IBB domain does not account for the Δ*iG* difference between importin α and importin β complexes (Fig. 2C).

A limitation of the present workflow is that it models binary Cap–importin interactions. The ternary α-β-cargo assembly is a more widely recognized configuration for nuclear transport receptors, and a full description would require modeling the ternary Cap–importin α-KPNB1 complex. We submitted the 3 sequences (Cap, importin α, and KPNB1) separately to the AlphaFold Server 3 times per combination, generating 15 models per complex, and compared all 3 interfaces pairwise using DockQ with the CAPRI acceptable threshold. No consistently similar interface was identified across combinations (Fig. S16). We therefore did not use ternary complex predictions in the present analysis. The binary Cap–importin models should be interpreted with this limitation in mind.

In our AF3-predicted models, the IBB domain frequently adopted an open conformation, with no stable contacts with the ARM repeats. Both closed and open conformations were sampled by AF3, and the conformation itself showed no deterministic relationship with Δ*iG* (Fig. 9A and B).

**Fig. 9.**
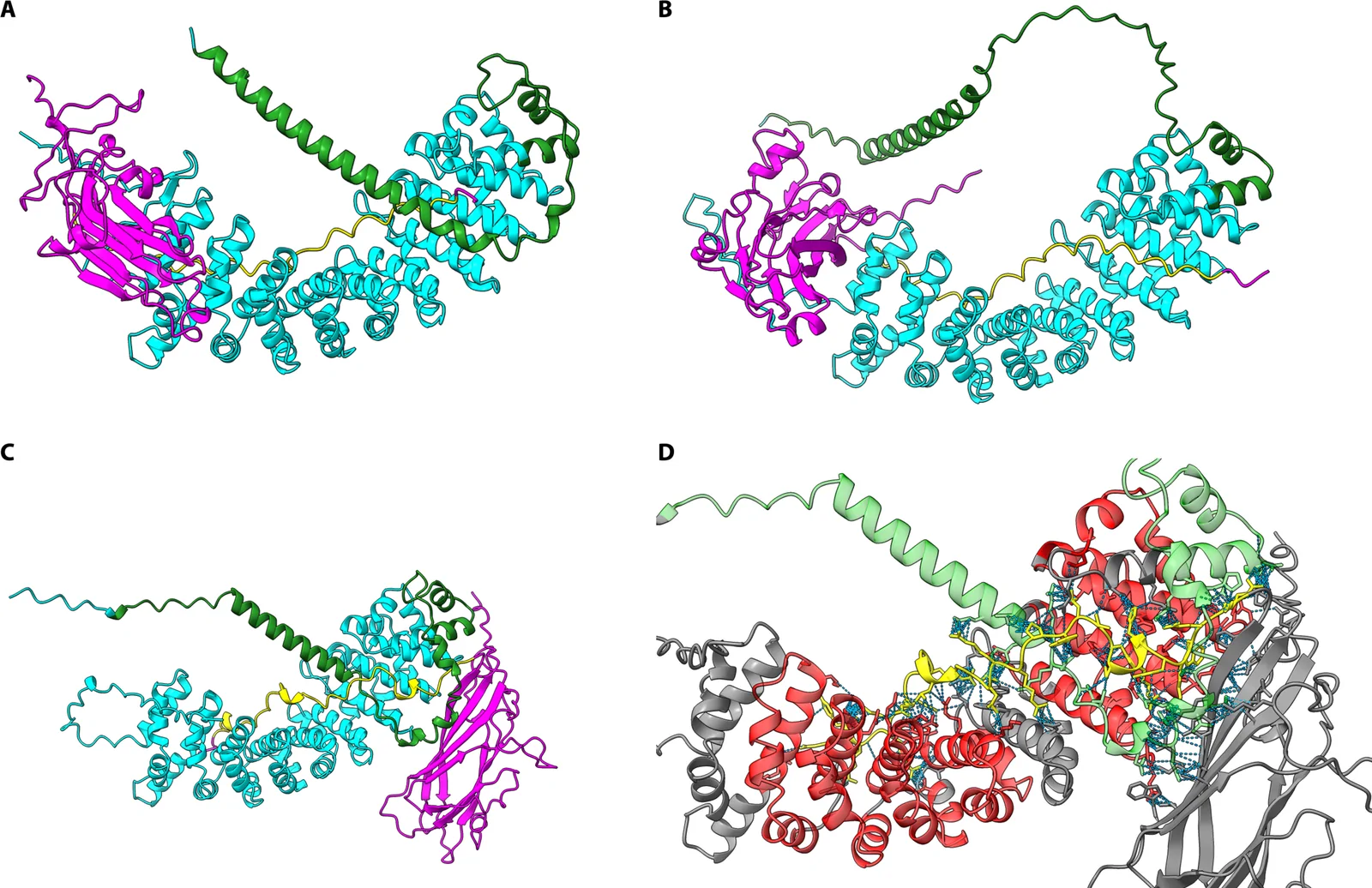
Structural features of predicted capsid protein (Cap)–importin α complexes illustrating conformational variability and nuclear localization signal (NLS) engagement. (A) Catfish circovirus (CCV) Cap–KPNA6 model 2 (Δ*iG* = 3.7 kcal/mol). (B) CCV Cap–KPNA6 model 3 (Δ*iG* = −14.3 kcal/mol). In both models, CCV Cap is shown in magenta, KPNA6 in cyan, the NLS region in yellow, and the importin β-binding (IBB) domain of KPNA6 in green. (C) Porcine circovirus type 2 (PCV2) Cap–KPNA1 complex. PCV2 Cap is shown in magenta, KPNA1 in cyan, the NLS region in yellow, and the IBB domain of KPNA1 in green. (D) Magnified view of the NLS–KPNA1 interface from (C). The ARM repeats of KPNA1 are highlighted in red, and contacts between the NLS and KPNA1 are indicated by blue dashed lines.

The open conformation is unlikely to be random noise. When importin α was predicted as a monomer, the autoinhibited conformation was consistently retained (Fig. S17), suggesting that the conformational change observed in the complex is associated with the presence of Cap. Moreover, several models with the IBB domain still in a partially occluding position exhibited notably low Δ*iG* values. For example, in the PCV2 Cap–KPNA4 model, the NLS was positioned between the IBB domain and the first 2 ARM repeats, suggesting potential competition between the NLS and the IBB domain for overlapping regions (Fig. 7B). This is reminiscent of influenza virus PB2, which can bind importin α directly and be imported into the nucleus by importin α alone [51]. In the PCV3 Cap–KPNA4 model, the IBB domain seemed to block the first 3 ARM repeats, yet the NLS still engaged the subsequent 5 ARM repeats. In other cases, the NLS appeared to be able to bypass the IBB domain entirely and bind to downstream ARM repeats from the opposite direction (Fig. 9C and D). These models all yielded low Δ*iG* values and might represent conformational states of importin α associated with NLS contacts.

These observations suggest that the higher Δ*iG* values of Cap–importin α complexes may not necessarily reflect a general structural tendency of circovirus capsids for importin β but could also arise from the energetic costs of conformational rearrangements, such as IBB displacement or bypass, that are not fully captured by static Δ*iG* calculations.

Relatively few studies have investigated cargo nuclear import mediated solely by individual importin β family members. Nevertheless, clear examples exist in virology. Parvoviruses, which are also small single-stranded DNA viruses with a broad host range similar to circoviruses, enter the nucleus via importin β [52].

Current domain prediction tools remain unreliable for most importin β group members. We therefore focused our structural analysis in the “Structural features of predicted NLS residues in Cap–importin complexes” section on KPNB1, which is relatively well studied. KPNB1 can function independently of importin α, and like many importin β group members, it typically recognizes cargo without relying on specific linear sequence motifs [53]. In our predicted Cap-KPNB1 models with low Δ*iG* values, the NLS regions predominantly engaged HEAT repeats 10 to 17. The N-terminal HEAT repeats 1 to 8, which are involved in RanGTP binding and cargo release, remained largely unoccupied—consistent with their additional role in binding FG-nucleoporins (Fig. 7A and Tables S9 to S11) [54].

As mentioned in Introduction, transportin belongs to the importin β superfamily but recognizes linear motifs. TNPO1 and TNPO2 recognize the PY-NLS motif, characterized by a C-terminal R-X_2–5_-P-Y epitope, whereas TNPO3 recognizes RS/RSY-rich motifs [55]. In our dataset, the lowest Δ*iG* value among all complexes was observed for the BCV Cap–TNPO1-X3 model (Δ*iG* = −25 kcal/mol), with similarly low Δ*iG* also seen for PCV2 TNPO2-X1 and ECV TNPO1.

However, the predicted NLS sequences of these viruses, R​RRY​RRR​RRR​ARP​MSH​LGA​LLR​RNP​SLA​APR​YRYRWRRKNG for BCV, PRRRYRRRRHRPRSHLGQILRRRPWLVHPRHRYRWRRK for PCV2, and DGPPARRARPNSASRRAFGKSSYSRVARIPRN for ECV, lack the consecutive P-Y residues characteristic of canonical PY-NLS motifs.

Notably, TNPO1 cargoes are not strictly limited to those carrying canonical PY-NLS motifs. For example, histone H3 binds TNPO1 with high affinity despite lacking the classical P-Y dipeptide, and this binding has been mapped to HEAT repeats 8 to 20 of TNPO1 [56]. This has been attributed to sufficient basic/hydrophobic interactions at epitope 1 of the NLS, while epitope 2 provides the R-X_2–5_-P-Y motif.

We therefore examined whether similar binding modes could be identified in our Cap-TNPO1 models. In the BCV and ECV models, the NLS regions were found to bind within HEAT repeats 11 to 18 and 11 to 16 of TNPO1, respectively, overlapping with the binding region reported for histone H3 (Fig. 7A and Tables S13 and S14). This raises the possibility that some circovirus capsid proteins may carry PY-NLS variants that, while lacking the canonical P-Y dipeptide, still engage transportin through a similar mode of recognition.

While predicted binding does not equate to functional nuclear import, it is not unprecedented for viral proteins to utilize multiple importins and enter the nucleus through distinct pathways. For example, the HIV-1 Rev protein has been shown to interact with and be imported by multiple nuclear transport receptors, including KPNB1, IPO5, IPO7, and TNPO1 [57]. Moreover, the import pathways used by Rev can vary across different cell types, highlighting the importance of cell- and tissue-specific transcriptomic data for a comprehensive assessment of which importins are actually available for viral exploitation in relevant target cells [58].

Taken together, these observations suggest that circovirus capsid proteins may be able to engage multiple importin groups through different binding modes: extended flexible NLSs for importin α, direct binding to importin β group members, and PY-NLS-like recognition for TNPO1.

### Host-associated importin binding patterns and their potential relationship to circovirus evolution

Among the 3 host classes, avian circoviruses displayed the most distinctive pattern. Avian circoviruses showed higher mean Δ*iG* for Cap–importin α complexes than mammalian and fish circoviruses (Fig. 3C). Avian-origin viral proteins may exhibit biochemical properties distinct from those of mammalian-origin viruses. In one study, replacement of a mammalian-adapted PB1 protein with its avian counterpart restored polymerase activity that had been suppressed at 40 °C, allowing replication at the normal avian body temperature of 42 °C [59]. In circoviruses, which have coevolved with their hosts for much longer periods, similar adaptive changes could have occurred.

Despite the high proportion of basic NLS residues and high positive charge at the avian Cap–importin interface, these features did not enable avian circoviruses to engage a broader range of importins (Fig. 6A to C). This paradox suggests that the relationship between NLS interface properties and importin usage is not simply driven by charge.

IPO11 was the top-ranked importin in mammalian circoviruses, while IPO9 showed the lowest mean Δ*iG* among all importins across the full dataset. Although PCV2 and PCV3 both infect pigs, their importin Δ*iG* profiles were distinct (Fig. 4D), suggesting that importin selectivity is determined by capsid protein properties rather than by host factors. Phylogenetic analyses showed that PCV2 and PCV3 have separate evolutionary origins, with PCV2 likely arising from bat circoviruses and PCV3 traced to rodent-associated lineages [60].

Fish circoviruses were predicted to engage the broadest range of importins among the 3 host classes, consistent with phylogenetic evidence identifying fish as the most ancient lineage within the *Circoviridae* family [60]. Circovirus-like sequences have been detected in fish chromosomes as endogenous viral elements, providing evidence of deep coevolution between circoviruses and their fish hosts [61].

### Circovirus capsid NLS interface properties, functional versatility, and evolutionary adaptability

The NLS is a stretch of basic amino acids and is therefore recognized by importins that are rich in acidic residues or, in other words, carry a net negative charge. However, the proportion of basic residues and the positive charge density were positively correlated with Δ*iG*. In other words, a higher proportion of basic residues was associated with weaker binding. Both linear and polynomial regression failed to capture the relationship, which prompted the use of SHAP analysis. SHAP dependence plots indicated that the association between NLS features and host-class discrimination was nonlinear and differed across host classes, with no single feature showing a consistent monotonic trend.

Structural information on circovirus Cap NLS–importin interfaces remains limited. To our knowledge, the only available experimental structure is the crystal structure of an isolated BFDV NLS peptide bound to mouse KPNA2 lacking the IBB domain (PDB: 4HTV; Fig. 10A) [23]. This peptide is shorter than the experimentally defined functional NLS region of BFDV, and only 7 residues of the peptide were resolved in the crystal structure. In addition, the structure was determined with a mouse importin α isoform. Because of these differences, a direct interface comparison (e.g., DockQ, interface root-mean-square deviation, or contact recovery) between this crystal structure and our full-length models was not performed. Nonetheless, both the crystal structure and the AF3-predicted BFDV Cap–KPNA2 model (Fig. 10B) show the NLS engaging the NLS-binding groove of KPNA2. In addition, as noted in the “Energetic and structural basis for importin family selectivity of circovirus capsids” section, the IBB domain did not prevent NLS binding to KPNA2 in the predicted model.

**Fig. 10.**
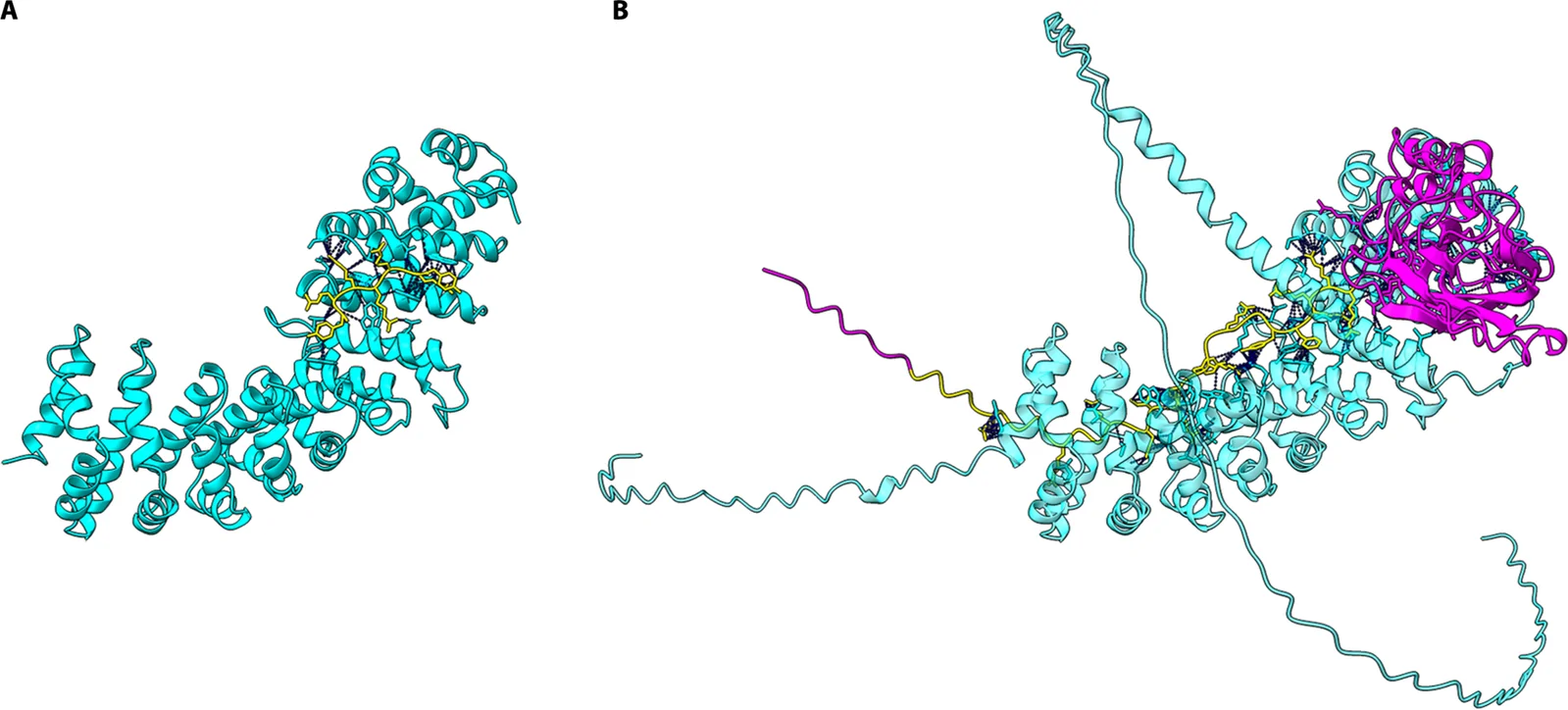
Structural illustration of beak and feather disease virus (BFDV) nuclear localization signal (NLS) binding to importin α. (A) Crystal structure of mouse KPNA2 (cyan) in complex with a BFDV NLS peptide (yellow) containing the core YRRRRRY motif (PDB: 4HTV). (B) AlphaFold 3 (AF3)-predicted model of full-length BFDV capsid protein (Cap; magenta) in complex with parrot KPNA2 (cyan); the predicted NLS region (residues 15 to 41) is shown in yellow, and blue dashed lines indicate contacts (interface predicted aligned error [iPAE] = 19.65).

In previous studies, when exogenous circovirus Cap proteins were expressed in eukaryotic cells, Cap was mainly localized to the nucleus, and Cap monomers were capable of self-assembling into viruslike particles [62,63]. The deletion of the NLS, however, indicated that this sequence not only facilitates monomeric Cap nuclear import but also participates in capsid assembly [64,65].

In the assembled circovirus particle, the NLS is oriented toward the interior of the capsid [19]. A similar scenario has been proposed for anelloviruses, where the C-terminal NLS is thought to have evolved in response to the progressive burial of the N-terminal NLS. To examine whether structural differences between the 2 viral families influence importin binding, we extended our structural analysis to a representative anellovirus capsid protein from porcine TTSV. Structural comparison of TTSV with PCV2 and PCV3 capsid monomers revealed a conserved N-terminal jelly-roll fold, but their importin binding modes differed markedly (Fig. S15A and B and Fig. 7B).

For importin α, we chose KPNA4 as a representative because both PCV2 and PCV3 yielded low-Δ*iG* models with this isoform (−7.4 and −11.0 kcal/mol, respectively). In the TTSV Cap–KPNA4 model shown in Fig. 7B, the C-terminal NLS appeared obstructed by the IBB domain. However, in other TTSV importin α models, such as TTSV Cap–KPNA2, the C-terminal NLS extended across the entire NLS-binding groove (Fig. S15C and D). Across most TTSV importin α models, the C-terminal NLS was consistently the primary binding interface. Notably, the interface similarity among TTSV Cap–importin α models was markedly higher than that observed for TTSV Cap–importin β models, a pattern not seen in the circovirus Cap–importin models. Moreover, although the dataset is limited, the majority of TTSV Cap–importin α models yielded negative Δ*iG* values, contrasting with the more variable trends observed for circoviruses (Fig. S15C and D).

For importin β, the patterns were also distinct. Unlike circovirus Cap–IPO9 complexes, which consistently had negative Δ*iG* values, the TTSV Cap–IPO9 model shown in Fig. 7B had a positive Δ*iG* (6.9 kcal/mol). This suggests that the Δ*iG* value is not simply determined by the importin family but also influenced by capsid architecture. In addition, while circovirus Caps engaged IPO9 through both the NLS and the surrounding capsid surface, TTSV Cap predominantly interacted with IPO9 through its 2 NLS regions, with limited involvement of the capsid body. In other TTSV Cap–importin β models, the NLS regions showed no consistent selection pattern, and most Δ*iG* values were positive (Fig. S15D).

The 2023 structural study interpreted the anellovirus ORF1 architecture as a circovirus-like jelly-roll core with a projection domain inserted between β-strands H and I [17]. As this projection domain increases in size, the C-terminal NLS also becomes longer, which was proposed to be associated with the progressive burial of the N-terminal NLS during capsid assembly [19]. This suggests that NLS evolution can be influenced by capsid structural changes. However, although the N-terminal NLS faces the interior of the assembled capsid in circoviruses, circoviruses have not evolved an additional NLS. Currently, evidence to explain how intact virions of circoviruses deliver their genome into the nucleus is insufficient. Some studies suggest that genome entry may occur without capsid disassembly, leaving open the question of whether intact virions enter the nucleus [66]. Additionally, circovirus capsids can undergo conformational changes inside host cells, so the possibility that such rearrangements expose basic amino acid clusters cannot be excluded [67].

The NLS features of viral proteins may undergo adaptive changes during evolution and even exhibit distinct patterns across different host species, as observed in other viruses. In polyomaviruses (PyVs), for instance, the NLSs of large tumor antigens (LTAs) exhibit marked evolutionary divergence that correlates with host species [68]. A systematic functional and structural analysis of human PyV LTAs revealed that their NLSs are remarkably heterogeneous in terms of structural organization, importin α isoform binding, and nuclear targeting abilities, despite all being located between the LXCXE motif and the origin-binding domain. This diversity is proposed to have arisen through duplication of a monopartite NLS and subsequent point mutations that optimized interactions with distinct importin α-binding sites.

Extending to nonmammalian hosts, the black sea bass PyV LTA was found to possess a bipartite classical NLS located downstream of its SF3 helicase domain—a position distinct from that in mammalian PyVs, which suggests that NLS position may vary with host species, reflecting virus–host adaptation [69]. These examples suggest that the host-associated features captured by our models may be linked to adaptive changes in NLS architecture, although deeper pattern characterization will require systematic sequence analysis of a larger panel of circovirus NLSs.

### Summary and limitations

In this study, we modeled 298 unique Cap–importin pairs covering different importin isoforms. For each pair, multiple AF3 conformational clusters were considered, and each representative conformation was further filtered by ZDOCK, yielding a total of 1,206 models (603 from AF3 and 603 from ZDOCK). We characterized each complex by its general interface properties as well as the specific features of its NLS at the binding interface. A random forest classifier was used for exploratory visualization to examine which NLS interface features were associated with host-class discrimination; this analysis was not used as evidence for predictive performance or generalizable relationships.

All 11 capsid proteins tested showed negative Δ*iG* for Cap–IPO9 complexes. The high evolutionary conservation of IPO9 across vertebrates is compatible with its identification as a candidate broadly interacting importin among the circoviruses examined.

Host-class-associated patterns were also observed in the predicted binding data. Fish circoviruses showed the broadest range of importins with low-Δ*iG* interactions and the highest proportion of low-Δ*iG* interactions. Mammalian circoviruses displayed more restricted, virus-specific profiles, as illustrated by the distinct Δ*iG* patterns of PCV2 and PCV3. Avian circoviruses showed a lower mean Δ*iG* for Cap–importin β complexes and a higher mean Δ*iG* for Cap–importin α complexes

NLS basic residue ratio, NLS interface occupancy, and NLS net charge differed across host classes (Fig. 6A to C). An exploratory machine learning analysis was consistent with these observations (Fig. 6D and E).

Several limitations should be considered. Most of the interactions described are computational predictions and have not been experimentally validated. Physical interaction was experimentally tested only for Cap–IPO9 across 3 virus–host pairs; all other Cap–importin combinations remain structural predictions. In addition, the Co-IP assays were performed in CHO-K1 cells, which are not the natural host cells of the tested viruses and therefore demonstrate physical association under the tested conditions, not functional nuclear import. The predicted models in our study only represent monomeric Cap and do not imply that intact virions or viruslike particles also have the potential to interact with importins. Moreover, the static structural models cannot capture the dynamic nature of the actual nuclear import process. Accordingly, this study is limited to the interaction level and does not address functional nuclear import. In addition, experimental structures of full-length circovirus Cap in complex with natural-host importins are currently unavailable, which limits direct structural benchmarking of the predicted interfaces.

In addition, Δ*iG* values were calculated using PDBePISA as a relative interface-solvation metric. These values are not experimentally measured binding affinities and may differ from physiological binding energetics. Molecular mechanics/generalized Born surface area was tested as an alternative, but its ΔTOTAL values were strongly linear with interface features (*R*^2^ = 0.816) (Fig. S18) [70,71].

The number of fish circoviruses examined is limited to 3, due to the scarcity of available sequences, which may affect the strength of the host-class-associated conclusions. The structural models are not fully independent, as multiple Cap–importin combinations share the same Cap sequence or the same importin. This residual dependence, together with the confounding between host class and viral lineage, may limit statistical power.

Therefore, the primary value of this study lies in providing a set of testable hypotheses for future experimental work, rather than establishing definitive biological mechanisms.

## Data Availability

All data supporting the findings of this study are available within the article and its Supplementary Materials. Structural models, confidence outputs, docking results, and derived tables have been deposited in Figshare (doi: 10.6084/m9.figshare.34009074).

## Supplementary Materials

Supplementary 1 Supplementary Methods Figs. S1 to S18 Tables S1 to S14 File S1 (folder: python script) Data Files S1 to S3 (file names: features_new.csv, Sequence ID.xlsx, PCV2_impa_IBB.xlsx)
