# Supplementary material for: Host-Associated Binding Patterns in Predicted Structural Models of Circovirus Capsid–Importin Complexes
Source: Comput Struct Biotechnol J. 2026 Nov 6;35(1):0253. doi: 10.34133/csbj.0253 (PMC13740329; doi:10.34133/csbj.0253)
Supplement: Supplementary file 1 [file csbj.0253.f1.zip › Supplementary Tables and Figures.docx]

### ****1 Supplementary Methods****

#### 1.1  **Molecular dynamics simulations**

To assess the dynamic stability of the predicted binding modes, we performed 50 ns molecular dynamics simulations on five representative Cap-importin complexes. Three complexes were selected as the highest ΔiG representatives from avian, mammalian, and fish hosts; two were selected as the lowest ΔiG representatives from avian and mammalian hosts. Simulations were performed using GROMACS 2025.3 with the AMBER99SB force field and TIP3P water model [32,33]. Each system was placed in a cubic box with a 1.5 nm solvent layer, neutralized with Na⁺/Cl⁻, and equilibrated at 300 K and 1 bar. Production runs were carried out for 50 ns with a 2 fs time step, using LINCS constraints, PME electrostatics with a 1.0 nm cutoff, and V-rescale temperature coupling. Backbone RMSD, per-residue RMSF, and interface hydrogen bond counts were analyzed over the trajectory.Results are provided in Supplementary Figures S2-S6.

#### 1.2  **Ternary complex prediction**

To assess whether the Cap-importin α-KPNB1 ternary complex could be modeled, we submitted the three sequences (Cap, importin α, and KPNB1) to the AlphaFold Server. For each combination, three random seeds were used, generating 15 models per complex. Each model was subjected to pairwise interface comparison across all three interfaces using DockQ with the CAPRI acceptable threshold. Representative ternary models are provided in Supplementary Figure S16. The comparison script is included as a commented section in Supplementary File S1 (compare.py).

#### 1.3  **MM-GBSA calculations**

MM-GBSA calculations were performed in two modes [71,72]. Single-frame calculations were applied to 206 Cap-importin complexes after energy minimization and NVT/NPT equilibration. Multi-frame calculations used 100 evenly spaced frames from the 50 ns production trajectories of the five representative complexes described in Section 1.1. All calculations used igb=2 and 0.15 M salt concentration. The resulting binding energies are provided in Data File S1. The relationship between MM-GBSA ΔTOTAL and interface features is shown in Supplementary Figure S18.

### ****2 Supplementary Tables****

#### Supplementary Table 1. AF3 confidence metrics by virus (mean±SD)

| **Virus** | **n** | **pLDDT** | **pTM** | **ipTM** | **iPAE** |
| --- | --- | --- | --- | --- | --- |
| GoCV | 44 | 76.000 ± 3.178 | 0.606 ± 0.047 | 0.223 ± 0.063 | 22.349 ± 3.721 |
| DuCV | 58 | 74.486 ± 2.547 | 0.580 ± 0.038 | 0.224 ± 0.088 | 23.052 ± 3.824 |
| BFDV | 47 | 74.393 ± 3.609 | 0.621 ± 0.051 | 0.289 ± 0.131 | 21.537 ± 3.411 |
| PiCV | 51 | 73.762 ± 3.170 | 0.601 ± 0.046 | 0.260 ± 0.097 | 22.495 ± 2.227 |
| PCV2 | 63 | 77.515 ± 2.633 | 0.634 ± 0.034 | 0.253 ± 0.066 | 21.728 ± 3.06 |
| PCV3 | 54 | 77.474 ± 2.776 | 0.638 ± 0.037 | 0.263 ± 0.095 | 22.579 ± 3.391 |
| HCV1 | 59 | 75.945 ± 4.137 | 0.626 ± 0.069 | 0.258 ± 0.111 | 21.109 ± 4.193 |
| BCV | 73 | 74.705 ± 3.015 | 0.596 ± 0.057 | 0.222 ± 0.078 | 22.779 ± 2.825 |
| ECV | 48 | 77.144 ± 2.183 | 0.621 ± 0.037 | 0.248 ± 0.062 | 22.225 ± 3.157 |
| BCV1 | 50 | 74.883 ± 3.577 | 0.627 ± 0.046 | 0.261 ± 0.109 | 22.711 ± 3.207 |
| CCV | 56 | 75.222 ± 3.085 | 0.640 ± 0.046 | 0.254 ± 0.103 | 22.766 ± 2.932 |
| Total | 603 | 75.591 ± 3.339 | 0.617 ± 0.051 | 0.249 ± 0.094 | 22.312 ± 3.321 |

#### Supplementary Table 2. ZDOCK concordance filtering results by virus

| **Virus** | **Total** | **Passed** | **Failed** | **Pass rate** | **Fail rate** |
| --- | --- | --- | --- | --- | --- |
| GoCV | 52 | 44 | 8 | 84.6% | 15.4% |
| DuCV | 68 | 58 | 10 | 85.3% | 14.7% |
| BFDV | 51 | 47 | 4 | 92.2% | 7.8% |
| PiCV | 55 | 51 | 4 | 92.7% | 7.3% |
| PCV2 | 72 | 63 | 9 | 87.5% | 12.5% |
| PCV3 | 71 | 54 | 17 | 76.1% | 23.9% |
| HCV1 | 79 | 59 | 20 | 74.7% | 25.3% |
| BCV | 90 | 73 | 17 | 81.1% | 18.9% |
| ECV | 52 | 48 | 4 | 92.3% | 7.7% |
| BCV1 | 57 | 50 | 7 | 87.7% | 12.3% |
| CCV | 60 | 56 | 4 | 93.3% | 6.7% |
| Total | 707 | 603 | 104 | 85.3% | 14.7% |

#### Supplementary Table 3. ZDOCK concordance filtering results by importin group

| **Importin group** | **Total** | **Passed** | **Failed** | **Pass rate** | **Fail rate** |
| --- | --- | --- | --- | --- | --- |
| Importin α | 260 | 220 | 40 | 84.6% | 15.4% |
| Importin β | 356 | 305 | 51 | 85.7% | 14.3% |
| Transportin | 91 | 78 | 13 | 85.7% | 14.3% |
| Total | 707 | 603 | 104 | 85.3% | 14.7% |

#### Supplementary Table 4. Predicted NLS sequences in circovirus Cap proteins

| **Virus** | **Predicted NLS sequence** |
| --- | --- |
| GoCV | RARPRSLYSRRRRATNRRRRYRRRRLHIGRIRSK |
| DuCV | RGRSYRRAYRGRRKRRGLRRRFRRRRLRIARPRRR |
| BFDV | RRRARPRYRRRHIRRYRPRRRYFRRRR |
| PiCV | RRRRFRRRRAPIRRRRIRRRRTRLSRNIRGHRRSSRIYYFRLRRKDK |
| PCV2 | PRRRYRRRRHRPRSHLGQILRRRPWLVHPRHRYRWRRK |
| PCV3 | RHRAIFRRRPRPRRRRRHRRRYVRRKLFIRRP |
| HCV1 | RRRRPRPRRRARRFRRIRRRRIYIRRP |
| BCV | RRRYRRRRRRARPMSHLGALLRRNPSLAAPRYRYRWRRK |
| ECV | DGPPARRARPNSASRRAFGKSSYSRVARIPRN |
| BCV1 | RPKMAARKARSFRRRRPIRRRRRVVSRRR |
| CCV | RRRTFRRPIRRRMHRRTRGRRMIRRRSRRSRP |

#### Supplementary Table 5. Confidence metrics and NLS-interface PAE for the models shown in Figure 7

| **Model** | **pLDDT** | **pTM** | **ipTM** | **iPAE** |
| --- | --- | --- | --- | --- |
| BFDV-Cap-KPNA6 | 70.21 | 0.65 | 0.19 | 22.03 |
| PCV2-Cap-KPNA6 | 75.01 | 0.63 | 0.24 | 23.86 |
| CCV-Cap-KPNA6 | 72.14 | 0.62 | 0.18 | 24.94 |
| DuCV-Cap-KPNB1 | 79.62 | 0.63 | 0.16 | 24.65 |
| BCV-Cap-KPNB1 | 78.95 | 0.62 | 0.21 | 20.64 |
| BCV1-Cap-KPNB1 | 78.77 | 0.63 | 0.19 | 24.67 |
| PiCV-Cap-TNPO1 | 76.61 | 0.64 | 0.18 | 20.15 |
| BCV-Cap-TNPO1 | 79.52 | 0.65 | 0.27 | 19.16 |
| ECV-Cap-TNPO1 | 78.23 | 0.62 | 0.24 | 21.64 |
| PCV2-Cap-KPNA4 | 76.00 | 0.60 | 0.29 | 19.34 |
| PCV3-Cap-KPNA4 | 75.18 | 0.62 | 0.22 | 25.04 |
| TTSV-Cap-KPNA4 | 69.40 | 0.43 | 0.16 | 24.57 |
| PCV2-Cap-IPO9 | 79.19 | 0.66 | 0.38 | 21.00 |
| PCV3-Cap-IPO9 | 77.21 | 0.65 | 0.25 | 21.09 |
| TTSV-Cap-IPO9 | 72.00 | 0.51 | 0.18 | 23.54 |

#### Supplementary Table 6. Residue contacts between BFDV NLS and KPNA6 across ARM repeats

| **ARM repeat** | **Residue range on KPNA6** | **KPNA6 contacting residues** | **BFDV NLS contacting residues** |
| --- | --- | --- | --- |
| 1 | 142-183 | Glu 140, Ser 183, Gly 184 | Arg 39, Arg 38 |
| 2 | 185–225 | - | - |
| 3 | 227–268 | Asn 258, Arg 257, Trp 261 | Arg 40, Arg 40, Phe 37 |
| 4 | 271–310 | Tyr 307, Asp 300 | Arg 31, Arg 40 |
| 5 | 312–352 | Thr 352, Gly 353 | Arg 24 |
| 6 | 354–394 | Glu 384 | Arg 29 |
| 7 | 396–436 | Glu 426 | Arg 29 |
| 8 | 439–479 | Glu 475, Val 469 | Arg 19, Tyr 22 |
| 9(Atypical) | 493–541 | - | - |

#### Supplementary Table 7. Residue contacts between PCV2 NLS and KPNA6 across ARM repeats

| **ARM repeat** | **Residue range on KPNA6** | **KPNA6 contacting residues** | **PCV2 NLS contacting residues** |
| --- | --- | --- | --- |
| 1 | 99–139 | Gly 140 | Arg 10 |
| 2 | 143–180 | - | - |
| 3 | 185–224 | - | - |
| 4 | 237–266 | Glu 252 | Arg 9 |
| 5 | 269–307 | Thr 297, Gly 309, Thr 310 | Arg 14, Arg 25 |
| 6 | 310–350 | Thr 314, Ser 336, Asn 347 | Arg 25, Arg 14, Arg 25 |
| 7 | 354–391 | - | - |
| 8 | 395–435 | Lys 339 | Ser 17 |
| 9(Atypical) | 444–487 | - | - |

#### Supplementary Table 8. Residue contacts between CCV NLS and KPNA6 across ARM repeats

| **ARM repeat** | **Residue range on KPNA6** | **KPNA6 contacting residues** | **CCV NLS contacting residues** |
| --- | --- | --- | --- |
| 1 | 101–142 | Asn 139 | Arg 4 |
| 2 | 145–183 | - | - |
| 3 | 188–227 | Asn 217, Asn 224, Arg 227 | Arg 6, Thr 7 |
| 4 | 239–269 | Asp 259, Asp 269, Gly 270 | Arg 6, Arg 9, Arg 13, Arg 15 |
| 5 | 272–310 | Val 310, Thr 311, Gly 312, Asp 313, Asp 314, Arg 304 | Arg 15, Arg 18, Ile 12 |
| 6 | 316–353 | Thr 317, Ser 349, Asn 350, Ala 353, Gly 354, Trp 346 | Arg 18, Met 16, Arg 21, Arg 15 |
| 7 | 355–394 | Glu 385 | Arg 14 |
| 8 | 398–437 | - | - |
| 9(Atypical) | 452–502 | Glu 477, Asp 496 | Arg 23, Arg 34 |

#### Supplementary Table 9. Residue contacts between DuCV NLS and KPNB1 across HEAT repeats

| **HEAT repeat** | **Residue range on KPNB1** | **KPNB1 contacting residues** | **DuCV NLS contacting residues** |
| --- | --- | --- | --- |
| 10 | 415–460 | Glu 437 | Arg 24 |
| 11 | 461–506 | Asn 469, Glu 479 | Arg 29, Arg 20 |
| 12 | 507–552 | Ser 526, Glu 530, Glu 534 | Arg 26, Arg 20 |
| 14 | 599–644 | Glu 626 | Arg 22 |
| 15 | 645–690 | Asp 676 | Lys 15 |
| 16 | 691–736 | Ser 715 | Arg 11 |
| 17 | 737–782 | Glu 763, Glu 767 | Arg 14, Arg 11 |
| 18 | 783–828 | Ala 816, Cys 817 | Arg 7 |

#### Supplementary Table 10. Residue contacts between BCV NLS and KPNB1 across HEAT repeats

| **HEAT repeat** | **Residue range on KPNB1** | **KPNB1 contacting residues** | **BCV NLS contacting residues** |
| --- | --- | --- | --- |
| 1 | 1-46 | Glu 136, Asp 143, Glu 144, Asp 147, Asn 140 | Arg 36, Arg 40, Arg 38 |
| 5 | 185-230 | Asp 192, Asp 194, Asp 195 | His 19, Arg 26 |
| 7 | 277-322 | Glu 292, Glu 334 | Arg 15 |
| 8 | 323-368 | Glu 334, Asn 324 | Arg 15, Ser 18 |
| 9 | 369-414 | Glu 385 | Ala 14 |
| 10 | 415-460 | Ser 437 | Arg 13 |
| 11 | 461-506 | Glu 481, Asp 482, Met 485 | Arg 11, Arg 13, Arg 8 |
| 12 | 507-552 | Asp 531, Arg 534 | Arg 8, Arg 4 |
| 13 | 553-598 | Ser 570, Asp 574 | Arg 6 |
| 14 | 599-644 | Glu 615 | Arg 9 |
| 15 | 645-690 | Asp 679 | Arg 4 |

#### Supplementary Table 11. Residue contacts between BCV1 NLS and KPNB1 across HEAT repeats

| **HEAT repeat** | **Residue range on KPNB1** | **KPNB1 contacting residues** | **BCV1 NLS contacting residues** |
| --- | --- | --- | --- |
| 7 | 277-322 | Asp 292 | Arg 40 |
| 10 | 415-460 | Asp 437 | Arg 39 |
| 12 | 507-552 | Glu 534 | Lys 19 |
| 14 | 599-644 | Glu 626, Leu 629, Met 630 | Arg 21, Arg 24 |
| 15 | 645-690 | Gln 665, Leu 668 | Arg 21, Arg 25 |
| 16 | 691-736 | Ser 715 | Arg 27 |
| 17 | 737-782 | Tyr 752, Asp 756, Glu 767 | Arg 32, Arg 27 |

#### Supplementary Table 12. Residue contacts between PiCV NLS and TNPO1 across HEAT repeats

| **HEAT repeat** | **Residue range on TNPO1** | **TNPO1 contacting residues** | **PiCV NLS contacting residues** |
| --- | --- | --- | --- |
| 4 | 135–165 | Asp164 | Arg30 |
| 5 | 180–220 | Glu167 | Arg27 |
| 7 | 262–291 | Glu281, Glu284, Ala290, Glu291 | Arg33, Arg22, Arg15, Arg19 |
| 8 | 378–396 | Asp372, Asp374, Thr375, Ser377 | Arg17, Arg21 |
| 9 | 403–443 | Glu432, Asp366, Asp368, Glu369 | Arg14, Arg20, Arg15, Arg16, Arg17 |
| 12 | 527–558 | Ile546, Asp549, Asp556 | Arg9, Arg7, Arg5 |
| 13 | 586–603 | Glu594 | Arg5, Arg7 |

#### Supplementary Table 13. Residue contacts between BCV NLS and TNPO1 across HEAT repeats

| **HEAT repeat** | **Residue range on TNPO1** | **TNPO1 contacting residues** | **BCV NLS contacting residues** |
| --- | --- | --- | --- |
| 11 | 481-521 | Ala505, Thr506, Glu509 | Arg13, Arg15 |
| 12 | 522-552 | Asp543, Thr547, Asp550 | Arg13, Arg15 |
| 13 | 579-597 | Asp582, Glu588, Ser591, Thr595 | Arg4, Arg8, Arg11, Arg34 |
| 14 | 638-655 | Asp639, Ser649, Glu653 | Arg4, Arg6, Arg8, Arg9, Arg11 |
| 15 | 669-708 | Gln685, Asp693 | Arg6, Tyr7 |
| 16 | 709-749 | Asn726, Asn727 | Arg5, Tyr7 |
| 17 | 764-792 | Thr766, Asn770 | Arg5, Tyr7 |
| 18 | 806-834 | Glu809 | Arg5 |

#### Supplementary Table 14. Residue contacts between ECV NLS and TNPO1 across HEAT repeats

| **HEAT repeat** | **Residue range on TNPO1** | **TNPO1 contacting residues** | **ECV NLS contacting residues** |
| --- | --- | --- | --- |
| 11 | 479-512 | Thr506, Glu509 | Arg13, Arg15 |
| 12 | 520-552 | Tyr542, Asp543, Asp550 | Arg13, Arg15 |
| 13 | 579-597 | Glu588, Ser592 | Arg4, Arg8, Arg11, Arg34 |
| 14 | 638-654 | Asp646 | Arg4, Arg6, Arg8, Arg9, Arg11 |
| 15 | 669-698 | Asp693 | Arg5, Tyr7 |
| 16 | 707-739 | Asn726 | Arg5 |

### ****3 Supplementary Figures****

#### ****Figure S1. Workflow and data hierarchy.**** 298 unique Cap-importin pairs were modeled with AF3 and grouped into 707 conformational clusters. After ZDOCK concordance filtering, 603 clusters passed (104 failed). Each passing cluster yielded one representative interface, represented by one AF3 model and one ZDOCK model, giving 603 AF3+603 ZDOCK=1,206 structural models.

A	B

C	C

E

#### ****Figure S2. Molecular dynamics simulation of the PCV3‑IPO4‑ZDOCK complex (PISA ΔiG=19.9 kcal/mol) over 50ns.**** (A) Initial structure at t=0ns, with the complex shown in cartoon representation (PCV3 Cap in magenta, IPO4 in cyan). (B) Final structure at t=50ns, showing that the overall binding mode is maintained. (C) Number of protein hydrogen bonds over the simulation trajectory. (D) Backbone RMSD of the complex relative to the initial structure over time. (E) Per‑residue RMSF calculated over the 50ns trajectory.

A	B

C	C

E

#### ****Figure S3. Molecular dynamics simulation of the BFDV‑IPO13-X1‑AF3 complex (PISA ΔiG=20.3 kcal/mol) over 50ns.**** (A) Initial structure at t=0ns, with the complex shown in cartoon representation (BFDV Cap in magenta, IPO13 in cyan). (B) Final structure at t=50ns, showing that the overall binding mode is maintained. (C) Number of protein hydrogen bonds over the simulation trajectory. (D) Backbone RMSD of the complex relative to the initial structure over time. (E) Per‑residue RMSF calculated over the 50ns trajectory.

A	B

C	C

E

#### ****Figure S4. Molecular dynamics simulation of the CCV‑IPO4‑ZDOCK complex (PISA ΔiG=15.6 kcal/mol) over 50ns.**** (A) Initial structure at t=0ns, with the complex shown in cartoon representation (CCV Cap in magenta, IPO4 in cyan). (B) Final structure at t=50ns, showing that the overall binding mode is maintained. (C) Number of protein hydrogen bonds over the simulation trajectory. (D) Backbone RMSD of the complex relative to the initial structure over time. (E) Per‑residue RMSF calculated over the 50ns trajectory.

A	B

C	C

E

#### ****Figure S5. Molecular dynamics simulation of the DuCV‑IPO9‑ZDOCK complex (PISA ΔiG=-18.7 kcal/mol) over 50ns.**** (A) Initial structure at t=0ns, with the complex shown in cartoon representation (DuCV Cap in magenta, IPO9 in cyan). (B) Final structure at t=50ns, showing that the overall binding mode is maintained. (C) Number of protein hydrogen bonds over the simulation trajectory. (D) Backbone RMSD of the complex relative to the initial structure over time. (E) Per‑residue RMSF calculated over the 50ns trajectory.

A	B

C	C

E

#### ****Figure S6. Molecular dynamics simulation of the BCV‑TNPO1-X3‑ZDOCK complex (PISA ΔiG=-25.0 kcal/mol) over 50ns.**** (A) Initial structure at t=0ns, with the complex shown in cartoon representation (BCV Cap in magenta, TNPO1 in cyan). (B) Final structure at t=50ns, showing that the overall binding mode is maintained. (C) Number of protein hydrogen bonds over the simulation trajectory. (D) Backbone RMSD of the complex relative to the initial structure over time. (E) Per‑residue RMSF calculated over the 50ns trajectory.

#### ****Figure S7. Paired comparison of ΔiG from AF3 and ZDOCK models.**** (A) Scatter plot of paired ΔiG values from AF3 and ZDOCK. Each point represents one conformation, with the red dashed line indicating y=x (perfect agreement). (B) Distribution of paired differences (AF3-ZDOCK). The red dashed line marks zero difference, and the blue solid line indicates the mean difference (0.22 kcal/mol). (C) Paired connection plot for the first 30 of 603 conformations, with gray lines connecting AF3 and ZDOCK predictions for the same conformation. (D) Bland-Altman plot comparing the two methods. The red solid line represents the mean bias (0.22 kcal/mol), and the blue dashed lines indicate the 95% limits of agreement (-8.06 to 8.51 kcal/mol). Paired comparisons were evaluated using the Wilcoxon signed-rank test (n=603, *P*=0.126).

#### ****Figure S8. Assessment of normality for ΔiG values.**** (A) Histogram of ΔiG values overlaid with a normal distribution curve fitted using the sample mean and standard deviation. (B) Quantile-quantile (Q-Q) plot comparing observed ΔiG quantiles against theoretical normal quantiles. (C) Box plot showing the median (center line), interquartile range (box), and whiskers extending to 1.5× the interquartile range. (D) Violin plot displaying the kernel density estimation of ΔiG values, with the red and black lines indicating the mean and median, respectively. Normality assessment yielded mixed results: the Shapiro-Wilk (*P*=3.70×10⁻⁴), D'Agostino-Pearson (*P*=1.89×10⁻³), and Anderson-Darling tests (rejected at the 5% level) indicated significant deviation from normality, whereas the Kolmogorov-Smirnov test did not (*P*=0.133). The distribution showed mild negative skewness (-0.24) and slightly low kurtosis (-0.16), suggesting an approximately symmetric but formally non-normal distribution. Accordingly, non-parametric tests were used for downstream analyses.

#### ****Figure S9. Mapping of predicted NLS regions and binding interface residues onto circovirus capsid proteins.**** For each of the 11 circovirus species, the full-length capsid protein sequence is shown as a light blue bar. The NLS regions predicted by NLStradamus are shown in red; residues identified as part of the Cap-importin interface by PDBePISA in representative AF3‑predicted Cap-IPO9 models are shown in green. Overlapping regions—where NLS residues are also located at the predicted interface—are shown in yellow. These overlapping residues correspond to the NLS interface residues used to calculate the NLS-related features in the dataset.

A	B	C	D

E	F

G	H

#### ****Figure S10. Full-length immunoblots corresponding to the Co-IP validation in Figure 1C-E.** (A-D) GoCV (avian): (A) Input, immunoblotted with anti-His. (B) Flag immunoprecipitation, IB with anti-His. (C) Input, IB with anti-Flag. (D) IP: Flag, IB with anti-Flag. (E-F) HCV1 (mammalian): (E) Input and IP: Flag, IB with anti-His. (F) Input and IP: Flag, IB with anti-Flag. (G-H) CCV (fish): (G)Input and IP: Flag, IB with anti-His. (H) Input and IP: Flag, IB with anti-Flag. Cap proteins were His-tagged and IPO9 was Flag-tagged. Representative blots from three independent experiments are shown. These experiments demonstrate physical association under the tested conditions and do not establish functional nuclear import.**

#### ****Figure S11. Importin-level sensitivity analysis of predicted ΔiG across importin groups.** Each importin is represented by its mean ΔiG across all circovirus Cap combinations. Bars indicate the mean across importins within each group, error bars indicate standard deviation, and black dots represent individual importins. The same trend as in the main text (Figure 2A) is observed: Cap-importin β complexes show the lowest mean ΔiG, followed by transportin and importin α.**

A	B

C	D

#### ****Figure S12. Virus-level and importin-level sensitivity analyses of predicted ΔiG and interface area.** (A-C) Virus-level sensitivity analyses corresponding to Figure 3A-C. Each virus is represented by its mean across all importin partners. Bars show the mean across viruses within each host class; error bars show standard deviation; black dots represent individual viruses. (A) Mean ΔiG by host class. (B) Mean interface area by host class. (C) Mean ΔiG with importin α by host class. The trends are consistent with Figure 3A-C. (D) Importin-level sensitivity analysis for avian viruses, corresponding to Figure 3D. Importin α shows a slightly higher mean ΔiG than transportin, which differs in direction from Figure 3D; however, these two groups did not differ significantly in the main text.**

#### ****Figure S13. Distribution of ΔiG values across all unique Cap-importin combinations.** Histogram shows the distribution of ΔiG values (n=298). Dashed lines indicate thresholds of -8, -10, and -12 kcal/mol, corresponding approximately to the 10th, 5th, and 3rd percentiles of the distribution, respectively. These thresholds were used to assess the sensitivity of host-class comparisons in Figure 5A.**

A	B	C

#### ****Figure S14. Virus-level sensitivity analysis of NLS interface features across host classes.**** (A) NLS interface occupancy. (B) NLS basic residue ratio. (C) NLS net charge. Bars show virus-level means, where each virus is represented by its mean across all importin partners (n=4 avian, 4 mammalian, 3 fish). Error bars indicate standard deviation across viruses within each host class, and individual dots represent individual viruses. Because of the small number of viruses per host class, formal statistical testing was not performed at this level; the direction of the differences was consistent with the combination-level analysis in Figure 6A-C.

A	B

C

D

#### ****Figure S15. Structural comparison of capsid proteins between circoviruses and torque teno sus virus (TTSV).**** TTSV capsid is shown in green; PCV2 capsid in magenta; PCV3 capsid in yellow. (A) Superposition of PCV2 and TTSV capsid monomer models. (B) Superposition of PCV3 and TTSV capsid monomer models. (C) AF3-predicted models of TTSV Cap with various importinα isoforms. (D) AF3-predicted models of TTSV Cap with importinβ family members. The N‑terminal NLS is shown in yellow, and the C‑terminal NLS in pink. Contacts between the NLS and importin are indicated by dark blue dashed lines to illustrate NLS engagement at the interface. The ΔiG values shown in parentheses are for the specific model displayed, not averaged values. The IPO11 model is not included because no ZDOCK pose similar to the AF3 prediction was identified for this combination. All models shown have passed ZDOCK concordance filtering. For complexes where multiple AF3 conformational clusters were identified (KPNA6, KPNB1, IPO7, and IPO13), the top-ranked AF3 model is shown.

A

B

#### ****Figure S16. AlphaFold3-predicted ternary complexes of circovirus capsid protein, importin α1 and KPNB1.**** (A) Six representative models for PCV2 Cap-importin α1-KPNB1. (B) Six representative models for GoCV Cap-importin α1-KPNB1. Capsid protein is shown in magenta, importinα1 in cyan, and KPNB1 in orange. The lack of a consistent binding interface across the six models for each virus illustrates the instability of AF3 ternary complex predictions for this system.

A

B

#### ****Figure S17. AF3-predicted structures of human importinα isoforms (KPNA1-7).**** (A) Monomer structures of KPNA1-7. In all seven isoforms, the IBB domain is folded back onto the ARM repeat domain, consistent with the autoinhibited conformation. (B) Comparison of representative importinα structures: monomeric form (orange, IBB in red) versus the same importinα extracted from a Cap-importinα complex model (cyan, IBB in grey). The IBB domain adopts a clearly distinct position in the complex-derived structure.

A

B	C	D

E

#### ****Figure S18. Comparison of PDBePISA and MM‑GBSA energy metrics and their relationship with interface features.**** (A) Scatter plots showing the relationship between single‑frame MM‑GBSA ΔTotal and interface features: interface area, hydrogen bonds (HB), and salt bridges (SB). Dashed lines represent linear regression fits; shaded bands are not shown. Linear regression coefficients and R² values are indicated for each feature. (B) Predicted versus actual single‑frame MM‑GBSA ΔTotal values from the multiple linear regression model: ΔTotal=28.59+(-0.0423)×Area+(-1.42)×HB+(-1.39)×SB (R²=0.816). (C) Validation of the single‑frame model on multi‑frame MM‑GBSA data. Multi‑frame ΔTotal values are plotted against values predicted by the single‑frame regression model. (D) Prediction interval validation using the shifted single‑frame model. All five multi‑frame MM‑GBSA values fall within the shifted prediction band, confirming that multi‑frame calculations follow the same linear relationship as single‑frame calculations but with a systematic offset. (E) Scatter plots showing the relationship between PDBePISA ΔiG and the same interface features: interface area, hydrogen bonds, and salt bridges. In contrast to MM‑GBSA, PDBePISA ΔiG shows no significant correlation with these features (R^2^ values near zero), indicating that PDBePISA captures a different aspect of binding energetics—primarily the solvation‑driven hydrophobic effect—rather than simply recapitulating interface geometry.
